# Epidemiology of cervical cancer in elderly women: Analysis of incidence, treatment, and survival using German registry data

**DOI:** 10.1002/cam4.6318

**Published:** 2023-07-05

**Authors:** Sonja Neumeyer, Luana Fiengo Tanaka, Linda A. Liang, Stefanie J. Klug

**Affiliations:** ^1^ Chair of Epidemiology, Department of Sport and Health Sciences Technical University of Munich Munich Germany

**Keywords:** cervical cancer, incidence, prognosis, survival, therapy

## Abstract

**Background:**

Cervical cancer (CC) screening is generally recommended until age 65. The incidence of CC could be underestimated, particularly in older women, due to a lack of hysterectomy correction. Furthermore, elderly women (≥65 years) are more often diagnosed with late‐stage disease and have worse outcomes than younger patients. This study aims to provide an in‐depth overview of CC in Germany.

**Methods:**

Incidence rates of CC (ICD‐10 C53) were determined using data from the German Centre of Cancer Registry data (ZfKD) of six federal state registries. Incidence was corrected by using hysterectomy prevalence values from a real‐world study. The distribution of treatment modalities (surgery, chemotherapy, radiation therapy) was assessed. Relative survival was calculated using the period approach (2011–2015). Survival was stratified by tumor (T) stage and histological type.

**Results:**

In total, 14,528 CC cases were included, 27.6% of which occurred in elderly women. Cumulative (2001–2015) age‐standardized incidence rates were 12.5 per 100,000 women without hysterectomy correction and 15.5 per 100,000 women after hysterectomy correction (+24% relative change). A lower proportion of elderly women were treated, especially in advanced tumor stages. Younger women (20–64 years) had a higher 5‐year relative survival compared to elderly women: 76.7% versus 46.9%, respectively. Survival was worse with increasing stage and for glandular histological subgroups, particularly among elderly women.

**Conclusions:**

CC incidence in elderly women is underestimated and survival is lower compared to younger women in Germany. Due to the high disease burden in elderly women, screening and treatment strategies need to be improved.

## 1 INTRODUCTION

Cervical cancer (CC) is the fourth most common cancer and the fourth leading cause of cancer mortality among women worldwide.^1^ In many high‐income countries (HIC) such as Germany, the incidence of cervical cancer has declined over time, mainly due to the introduction of cytological screening in the 1960s and 1970s. To date, the incidence of CC in Germany is somewhat higher compared to other HIC,^2^, ^3^ with an age‐standardized incidence rate of 8.6 per 100,000 women (old European standard) in 2018.^3^ However, incidence peaks at 16 per 100,000 for ages 40–44 and ages 60–64, and varies between 12 and 14 per 100,000 for women aged ≥65. Women aged 65 and older constitute around 30% of all cases of CC in Germany and 50% of all deaths due to this malignancy. This may indicate potential issues with screening.

In many countries with organized screening, screening for CC is only recommended up to the age of 65 years.^4^, ^5^, ^6^ First, this is generally due to the higher incidence in women of reproductive age (<50 years), where the risk of developing CC decreases linearly from 0.6% at age 45 to 0.2% at ages 75 and above^7^ and second, due to the successful detection of at‐risk cases in the prior years within well‐organized screening programs where older women tend to be well‐screened.^8^ From 1971 to 2019, Germany had an opportunistic CC screening system starting at age 20 years without an upper age limit for yearly pap smears. Women aged 40–69 years were more likely to participate in screening than younger women (20–29 years) but no difference was observed for women older than 70 years.^9^ In 2020, Germany introduced an organized screening program where women between ages 20 and 65 receive letters from their statutory health insurance every 5 years with information about the program.^10^ This new program was implemented to improve screening effectiveness. After age 65, the continuation of screening is to be decided by the physician.

The burden of CC is likely underestimated particularly in elderly women, due to the utilization of the general female population as the denominator in incidence calculations, by failing to acknowledge that part of the female population no longer remains at risk for CC after undergoing total hysterectomy. Hysterectomies are also not exclusive to older age. In Germany, 1.2% of women aged 30–34 years, 21.5%–34% of women aged 50–59 years, and 40.7% of women aged 60–64 years underwent hysterectomy.^11^ Incidence studies, which have accounted for hysterectomies in Germany and the United States (US), show that CC incidence is highly underestimated in elderly women.^12^, ^13^, ^14^

Regarding prognosis and survival, 5‐year relative survival of CC ranges from 50% to 70% globally and has been increasing due to improvements to treatment approaches.^15^ In Europe, 5‐year relative survival is approximately 61% and decreases linearly from 85% among young women aged 15%–29% to 68% (women aged 45–54 years) and to 34% (elderly women ≥75 years).^16^ The same downward trend is observed for localized versus regional or metastatic cancer stages. Evidence from the US suggests that elderly cancer patients are managed differently^17^, ^18^, ^19^ and thus have worse outcomes compared to younger patients.^17^, ^18^ Furthermore, elderly women are more often diagnosed with advanced‐stage disease and are more likely to die prematurely from CC.^20^ There may be an unmet need among older women in terms of prevention and treatment of CC.

Therefore, the aim of the present study is to describe the epidemiology of CC in Germany. We calculated incidence trends with and without correction for hysterectomies, treatment modalities and survival of CC across all screen‐relevant age groups, with a focus on outcomes for elderly women, using national cervical cancer registry data.

## 2 METHODS

### 2.1 Data

The cumulative incidence rates 2001–2015 of CC (ICD‐10 C53) were calculated using data from the German Centre of Cancer Registry Data (ZfKD) at the Robert Koch Institute (RKI). Data of the following six German Cancer Registries were included in this analysis, with completeness of registration >90%^7^ and data available for the years 2001–2015: Hamburg, Mecklenburg‐Western Pomerania, Rhineland‐Palatinate, Saarland, Saxony and Schleswig‐Holstein. These federal states cover around 19% of the entire German population. The available data covered between 18.6% (in year 2004) and 20.5% (in year 2013) of CC detected in all of Germany. ZfKD data do not contain any sociodemographic data such as education level but do provide tumor‐related information such as histological group, stage, grading, and general treatment approaches.

We obtained cases aged 20 and older (*n* = 14,528). Women with in situ carcinoma (*n* = 9) were excluded. Patients with diagnosis confirmed via death certificate only (DCO) (*n* = 835 DCO cases (5.4%), with *n* = 170 (1.6%) for women <65 years and *n* = 665 (14.2%) for women ≥65) were excluded from the main analyses. Elderly age in this study is defined as 65 years and older and compared to younger women (20–64 years of age). Population data for Germany were obtained from the German Federal Statistical Office.^21^

### 2.2 Statistical analyses

The descriptive analyses included the absolute number of CC cases for all age groups (20–34, 35–49, 50–64, 65–74, 75–84, ≥85 and age groups <65 and ≥65), the proportion of elderly women among all CC diagnoses, the distribution of main histological types (squamous cell carcinoma, adenocarcinoma, and adenosquamous carcinoma)^22^ in elderly women (vs. younger women), the distribution of tumor (T) stage at diagnosis (Stage 1/local, Stage 2/regional, Stage 3/regional, and Stage 4/distant), the distribution of tumor grading (well‐differentiated, moderately differentiated, poorly differentiated, and undifferentiated), the mean age at diagnosis by histological group, and the distribution of treatment modalities (surgery, chemotherapy, and radiation therapy) in elderly women (vs. younger women). Treatment is defined as any treatment with either surgery, chemotherapy, or radiation versus never receiving any treatment.

Age‐standardized and age group‐specific incidence rates (30–39, 40–49, 50–59, 60–69, and 70–79) were calculated both as uncorrected and corrected for hysterectomy prevalence. Incidence rates were age‐standardized by the old European standard population,^23^ the world population proposed by Segi and modified by Doll^24^ and the standard 2000 US population.^25^ For hysterectomy correction, the population at risk (denominator) was corrected by removing the proportion of women with a reported hysterectomy. Hysterectomy prevalence values were taken from the DEGS1 study (German Health Interview and Examination Survey for adults) (hysterectomy correction 1).^26^ For better comparison with official incidence statistics by the RKI or SEER (Surveillance, Epidemiology, and End Results Program), we calculated incidence rates using case counts and population data for all age groups (including <20 years of age).

To assess incidence changes over time among the different age groups for all cervical cancers combined, as well as by histological group, annual percent change (APC) and corresponding 95% confidence intervals (CI) were calculated using the default settings of Joinpoint software (Version 4.9.0.0, National Cancer Institute).^27^ This program uses log‐linear joinpoint regression models to fit straight lines to data points to detect possible time trends. Multiple APC values are shown in case statistically significant breakpoints were found.

One‐, 2‐, and 5‐year relative survival rates were calculated among women after a CC diagnosis. For these analyses, the period approach^28^, ^29^ was used, which provides more up‐to‐date estimates of long‐term cancer survival compared to traditional cohort‐based analysis. The present analysis includes the survival of patients in the time period from 2011 to 2015. Relative survival was calculated by dividing observed survival by expected survival in the general population derived from life tables for the population of Germany using the Ederer II method.^30^ Survival rates were stratified by T stage and histological group for all patients combined as well as for elderly and younger women. For the survival analyses, the R package periodR^31^ was used. Relative 5‐year survival curves for both age groups (elderly vs. young) and survival curves by stages at diagnosis were plotted using the R package periodR.

Multivariable Cox proportional hazard models were used to assess survival in elderly and younger women. Models were adjusted by factors that influence survival: T stage (Stage 1/local, Stage 2/regional, Stage 3/regional, and Stage 4/distant), histological group (squamous carcinoma, adenocarcinoma, adenosquamous carcinoma, and others/unspecified), and treatment (yes/no). Hazard ratios (HR) and corresponding 95% CI were reported. *P*‐values <0.05 were considered statistically significant. Missing data were excluded and available case analyses were carried out. These statistical analyses were performed using R Statistical Software (version 4.0.3; R Foundation for Statistical Computing).^32^

## 3 RESULTS

In total, 14,528 women diagnosed with cervical cancer between 2001 and 2015 were included in these analyses. Elderly women constituted 27.6% (*n* = 4015) of all cervical cancer cases of the study population (Table 1). The majority of patients had localized cancer (stage 1, 49.2%) and squamous cell carcinoma (72.9%).

**TABLE 1:** Distribution of tumor characteristics of cervical cancer cases (*N* = 14,528) according to age groups for the years 2001–2015.

	Age groups															Age groups			
	Total		Age^a^	20–34		35–49		50–64		65–74		75–84		≥85		<65		≥65	
	*N*	%	years	*N*	%	*N*	%	*N*	%	*N*	%	*N*	%	*N*	%	*N*	%	*N*	%
***N* cases (proportion per age group)**	14,528		54.5	1517	10.4	5025	34.6	3971	27.3	2046	14.1	1446	10.0	523	3.6	10,513	72.4	4015	27.6
**Histological group subtype**																			
Squamous carcinoma	10,585	72.9	53.7	1152	75.9	3756	74.7	2969	74.8	1444	70.6	943	65.2	321	61.4	7877	74.9	2716	67.6
Adenocarcinoma	2570	17.7	57.6	252	16.6	874	17.4	662	16.7	381	18.6	300	20.7	101	19.3	1788	17.0	787	19.6
Adenosquamous carcinoma	342	2.4	52.5	31	2.0	146	2.9	89	2.2	48	2.3	25	1.7	3	0.6	266	2.5	76	1.9
Others/unspecified	1031	7.1	61.0	82	5.6	249	5.7	251	9.2	173	14.2	178	25.8	98	45.0	582	5.5	436	10.9
**Tumor (T) stage**																			
Stage 1/local	7150	49.2		1194	78.7	3276	65.2	1625	40.9	653	31.9	330	22.8	72	13.8	6095	58.0	1055	26.3
Stage 2/regional	2802	19.3		134	8.8	784	15.6	961	24.2	521	25.5	314	21.7	88	16.8	1879	17.9	923	23.0
Stage 3/regional	1250	8.6		19	1.3	265	5.3	425	10.7	236	11.5	227	15.7	78	14.9	709	6.7	541	13.5
Stage 4/distant	854	5.9		21	1.3	160	3.2	307	7.7	186	9.1	133	9.2	47	9.0	488	4.6	366	9.1
Missing	2472	17.0		149	9.8	540	10.7	653	16.4	450	22.0	442	30.6	238	45.5	1342	12.8	1130	28.1
**Tumor grading**																			
Well‐differentiated	1049	7.2		198	13.1	466	9.3	226	5.7	86	4.2	58	4.0	15	2.9	890	8.5	159	0.4
Moderately differentiated	6277	43.2		645	42.4	2279	45.4	1770	44.6	865	42.3	532	36.8	186	35.6	4694	44.6	1583	39.4
Poorly differentiated	5058	34.8		398	26.2	1651	32.9	1497	37.7	778	38.0	561	38.8	173	33.1	3546	33.7	1512	37.7
Undifferentiated	120	0.8		9	0.6	23	0.5	30	0.8	28	1.4	21	1.5	9	1.7	62	0.6	58	1.4
Missing	2024	13.9		267	17.6	606	12.1	448	11.3	289	14.1	274	18.9	140	26.8	1321	12.6	703	17.5

^a^ Mean age at diagnosis.

Regarding histologic types by age groups, younger women <65 years were predominantly diagnosed with squamous carcinoma (74.9%) and adenocarcinoma (17.0%), whereas 67.6% of elderly women were diagnosed with squamous carcinoma and 19.6% with adenocarcinoma (Table 1). Younger women were more often diagnosed with Stage 1 cancer (58.0%) compared to elderly women (26.3%). Similarly, younger women were more often diagnosed with well or moderately differentiated tumors compared to elderly women. For a comparison of proportions including DCO cases, see Table S1.

The observed cumulative (2001–2015) and uncorrected age‐standardized incidence rate (European standard) for cervical cancer was 12.5 per 100,000 women (95% CI, 12.3–12.7; Table 2). After correction for hysterectomy, the incidence increased by 24% to 15.5 per 100,000 (95% CI, 15.3–15.8). Regarding age group‐specific rates, incidence (uncorrected) peaked for women in the age group 40–49 with 19.8 new cases per 100,000 women (Table 2). Relative changes after hysterectomy correction increased linearly and by 65.1% when comparing uncorrected with corrected incidence rates in the older age groups (age group 70–79 using the DEGS study). Hysterectomy‐corrected incidence rates increased in older women with 23.2 new CC cases per 100,000 women in those aged 70–79. Age‐standardized incidence rates using different standard populations by year are shown in Table S2, corrected and uncorrected for hysterectomy.

**TABLE 2:** Age‐specific incidence of cervical cancer per 100,000 women with and without correction for hysterectomized women in Germany from 2001 to 2015.

	Cumulative age‐standardized incidence rate—not corrected for hysterectomy (95% CI)	DEGS study: Hysterectomy prevalence (%)^a^	Incidence corrected for hysterectomy (95% CI)^b^	Relative change (%)^c^
**All ages**	12.5 (12.3–12.7)^d^	17.5%	15.5 (15.3–15.8)	24%
**Age groups (years)**				
30–39	16.9 (16.2–17.6)	0.8%	17.0 (16.4–17.7)	0.8%
40–49	19.8 (19.1–20.4)	10.9%	22.2 (21.5–23.0)	12.2%
50–59	17.3 (16.7–17.9)	27.5%	23.9 (23.0–24.8)	37.9%
60–69	15.2 (14.6–15.8)	32.4%	22.5 (21.6–23.4)	48.0%
70–79	14.1 (13.4–14.7)	39.4%	23.2 (22.1–24.3)	65.1%

^a^ Prevalence values taken from the DEGS1 study, Prütz et al. (2013).^11^

^b^ Population at risk reduced using hysterectomy prevalences by Prütz et al. (2013).^11^

^c^ Relative change comparing incidence corrected for hysterectomy using prevalence values of the DEGS study with the uncorrected incidence.

^d^ Age–standardized using the old European standard population.

Abbreviations: CI: confidence interval; DEGS: German Health Interview and Examination Survey for adults.

Trends in incidence rates are very similar for uncorrected and hysterectomy‐corrected rates, showing an overall decrease between 2001 and 2015 (Figure S1). For all age groups, the incidence rates decreased or were stable in the observation period from 2001 to 2015 (Figures S2 and S3), except for the age group 20–29. For this age group, the incidence rates increased significantly from 2001 to 2015 with an APC of 3.5% (95% CI: 1.5–5.7%) for corrected rates (Figure 3A). For the age groups 30–39 and 50 and above, incidence rates decreased significantly (Figure 3B, D‐F). Incidence rates were stable for the age group 40–49 at −0.6% (95% CI: −1.8%–0.7%; Figure S3C). Incidence rates by diagnosis year and histological group are shown in Table S3.

The distribution of treatment (surgery, chemotherapy, and radiation therapy) across age groups is shown in Table 3. In total, 42.6% of cervical cancer patients received any of the following treatments: surgery, chemotherapy, radiation therapy, and 4.0% received no treatment. A larger proportion of elderly women (8.9%) received no treatment compared to younger women (2.2%). For all T stages (1–4), a lower proportion of elderly women were treated with surgery or chemotherapy compared to younger women. For example, 14.8% of elderly women with T stage 4 cervical cancer were treated with surgery compared to 18.0% of younger women. Differences were even more pronounced for chemotherapy, with 13.1% of elderly T stage 4 patients being treated with chemotherapy compared to 34.6% of younger women. These differences apply specifically for women of the oldest age groups 75–84 and ≥85. In contrast, elderly women with T stage 1–3 cancers were more often treated with radiotherapy compared to younger women. However, for stage 4 cancers, the inverse was observed: younger women (45.1%) being treated with radiation therapy compared to 35.8% of elderly women. Table S4 shows the distribution of treatment modalities including DCO cases.

**TABLE 3:** Distribution of treatment uptake of cervical cancer cases according to age and tumor stage from 2001 to 2015 in Germany.

			Age groups												Age groups			
			20–34		35–49		50–64		65–74		75–84		>85		< 65		≥ 65	
	*N*	%	*N*	%	*N*	%	*N*	%	*N*	%	*N*	%	*N*	%	*N*	%	*N*	%
**Any treatment**																		
Yes	6182	42.6	660	43.5	2239	44.6	1688	42.5	928	45.4	528	36.5	139	26.6	4587	43.6	1595	39.7
No	585	4.0	22	1.5	81	1.6	124	3.1	105	5.1	150	10.4	103	19.7	227	2.2	358	8.9
Missing	7761	53.4	835	55.0	2705	53.8	2159	54.4	1013	49.5	768	53.1	281	53.7	5699	54.2	2062	51.4
**Treatment:**																		
**Surgery**																		
Stage 1																		
Yes	3308	46.3	563	47.2	1530	46.7	734	45.2	319	48.9	140	42.4	22	30.6	2827	46.4	481	45.6
No	145	2.0	17	1.4	43	1.3	30	1.8	25	3.8	21	6.4	9	12.5	90	1.5	55	5.2
Missing	3697	51.7	614	51.4	1703	52.0	861	53.0	309	47.3	169	51.2	41	56.9	3178	52.1	519	49.2
Stage 2																		
Yes	1007	35.9	51	38.1	305	38.9	341	35.5	209	40.1	83	26.4	18	20.5	697	37.1	310	33.6
No	340	12.1	3	2.2	55	7.0	111	11.6	77	14.8	65	20.7	29	33.0	169	9.0	171	18.5
Missing	1455	51.9	80	59.7	424	54.1	509	53.0	235	45.1	166	52.9	41	46.5	1013	53.9	442	47.9
Stage 3																		
Yes	173	13.8	3	15.8	51	19.2	56	13.2	30	12.7	30	13.2	3	3.8	110	15.5	63	11.6
No	497	39.8	3	15.8	80	30.2	168	39.5	109	46.2	103	45.4	34	43.6	251	35.4	246	45.5
Missing	580	46.4	13	68.4	134	50.6	201	47.3	97	41.1	94	41.4	41	52.6	348	49.1	232	42.9
Stage 4																		
Yes	142	16.6	8	38.1	35	21.9	45	14.7	34	18.2	17	12.8	3	6.4	88	18.0	54	14.8
No	369	43.2	4	19.0	58	36.3	137	44.6	77	41.2	70	52.6	23	48.9	199	40.8	170	46.4
Missing	344	40.2	9	42.9	67	41.9	125	40.7	76	40.6	46	34.6	21	44.7	201	41.2	142	38.8
**Chemotherapy**																		
Stage 1																		
Yes	538	7.5	79	6.6	268	8.2	139	8.6	46	7.0	6	1.8	0	0	486	8.0	52	4.9
No	2661	37.2	472	39.5	1178	36.0	564	34.7	276	42.3	143	43.3	28	38.9	2214	36.3	447	42.4
Missing	3951	55.3	643	53.9	1830	55.9	922	56.7	331	50.7	181	54.8	44	61.1	3395	55.7	556	52.7
Stage 2																		
Yes	654	23.3	36	26.9	218	27.8	269	28.0	112	21.5	19	6.1	0	0	523	27.8	131	14.2
No	617	22.0	18	13.4	119	15.2	156	16.2	158	30.3	122	38.9	44	50.0	293	15.6	324	35.1
Missing	1531	54.7	80	59.7	447	57.0	536	55.8	251	48.2	173	55.1	44	50.0	1063	56.6	468	50.7
Stage 3																		
Yes	329	26.3	3	15.8	101	38.1	146	34.4	63	26.7	14	6.2	2	2.6	250	35.3	79	14.6
No	344	27.5	3	15.8	29	10.9	80	18.8	75	31.8	121	53.3	36	46.2	112	15.8	232	42.9
Missing	577	46.2	13	68.4	135	50.9	199	46.8	98	41.5	92	40.5	40	51.3	347	48.9	230	42.5
Stage 4																		
Yes	217	25.4	11	52.4	63	39.4	95	30.9	38	20.4	10	7.5	0	0	169	34.6	48	13.1
No	299	35.0	2	9.5	34	21.3	86	28.0	74	39.8	77	57.9	26	55.3	122	25.0	177	48.4
Missing	338	39.6	8	38.1	63	39.4	126	41.0	74	39.8	46	34.6	21	44.7	197	40.4	141	38.5
**Radiation therapy**																		
Stage 1																		
Yes	917	12.8	95	8.0	379	11.6	248	15.3	119	18.2	68	20.6	8	11.1	722	11.8	195	18.5
No	2295	32.1	457	38.3	1069	32.6	459	28.2	206	31.5	82	24.8	22	30.6	1985	32.6	310	29.4
Missing	3938	55.1	642	53.8	1828	55.8	918	56.5	328	50.2	180	54.6	42	58.3	3388	55.6	550	52.1
Stage 2																		
Yes	938	33.5	38	28.4	252	32.1	321	33.4	209	40.1	95	30.3	23	26.1	611	32.5	327	35.4
No	344	12.3	16	11.9	87	11.1	106	11.0	63	12.1	50	15.9	22	25.0	209	11.1	135	14.6
Missing	1520	54.2	80	59.7	445	56.8	534	55.6	249	47.8	169	53.8	43	48.9	1059	56.4	461	50.0
Stage 3																		
Yes	535	42.8	4	21.1	113	42.6	183	43.1	103	43.6	102	44.9	30	38.5	300	42.3	235	43.4
No	141	11.3	2	10.5	18	6.8	42	9.9	35	14.8	33	14.5	11	14.1	62	8.7	79	14.6
Missing	574	45.9	13	68.4	134	50.6	200	47.1	98	41.6	92	40.5	37	47.4	347	49.0	227	42.0
Stage 4																		
Yes	351	41.1	11	52.4	73	45.6	136	44.3	66	35.5	54	40.6	11	23.4	220	45.1	131	35.8
No	168	19.7	2	9.5	25	15.6	47	15.3	46	24.7	33	24.8	15	31.9	74	15.2	94	25.7
Missing	335	39.2	8	38.1	62	38.8	124	40.4	74	39.8	46	34.6	21	44.7	194	39.8	141	38.5

Any treatment is defined as having received any of the treatments: surgery, chemotherapy, and radiation therapy.

**FIGURE 1:**
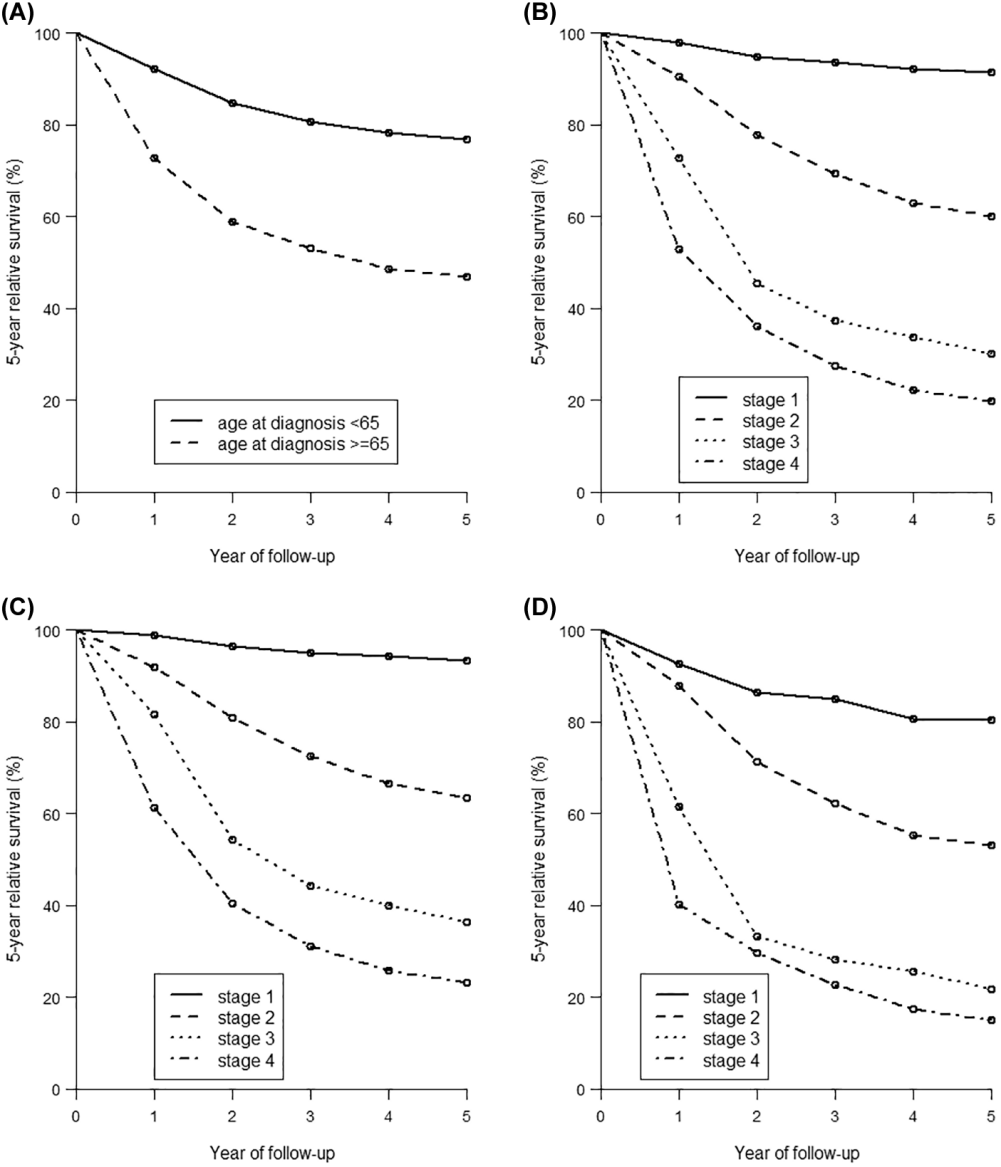
Relative survival curves comparing: (A) relative survival of women diagnosed with cervical cancer at ages <65 versus ages ≥65; (B) Survival curve by stage (all ages); (C) Survival curve of women <65 years by stage at diagnosis; (D) Survival curve of women ≥65 years by stage at diagnosis.

The overall 1‐, 2‐, and 5‐year relative survival rates among women with cervical cancer were 86.6%, 77.7%, and 68.6% (Table 4). Patients in the younger age group had a moderate 5‐year relative survival of 76.7% compared to 46.9% among elderly patients. The yearly relative survival rates differed substantially by stage at diagnosis (Figure 1B), from 91.4% for Stage 1 (local), 60.1% for Stage 2 (regional), 30.1% for Stage 3 (regional), and 19.7% for Stage 4 cancers (distant) for 5‐year survival. Women diagnosed with Stage 1 cervical cancer had 5‐year relative survival rates of 93.4% in the age group <65 years (Figure 1C) and 80.4% in the age group ≥65 years (Figure 1D). These 5‐year survival rates decreased with increasing disease stage: with 23.2% relative survival in younger women (<65 years) and 14.9% in older women with Stage 4 cervical cancer. In both age groups and overall, women diagnosed with adenosquamous carcinoma had the poorest 5‐year relative survival (Table 4). Comparing histological group subtypes, younger women had the highest 5‐year relative survival when diagnosed with adenocarcinoma compared to older women who had the highest relative survival when diagnosed with squamous carcinoma.

**TABLE 4:** One, 2, and 5‐year relative survival of patients with cervical cancer, and the 5‐year relative survival by age groups, tumor stage, and histological group subtype in Germany from 2001 to 2015.

	Overall		Age group <65		Age group ≥65	
	*N*	% (95% CI)	*N*	% (95% CI)	*N*	% (95% CI)
**Relative survival**						
1‐year survival	8350	86.6 (85.8–87.8)	6254	92.2 (91.2–93.2)	2096	72.6 (70.1–75.1)
2‐year survival	8350	77.7 (76.5–78.9)	6254	84.7 (83.5–85.9)	2096	58.6 (55.7–61.5)
5‐year survival	8350	68.6 (67.2–70.0)	6254	76.7 (75.1–78.3)	2096	46.9 (43.8–50.0)
**5‐year survival by tumor (T) stage at diagnosis**						
Stage 1/local	4507	91.4 (90.0–92.8)	3832	93.4 (92.2–94.6)	675	80.4 (74.7–86.1)
Stage 2/regional	1606	60.1 (56.6–63.6)	1091	63.4 (59.5–67.3)	515	53.2 (46.3–60.1)
Stage 3/regional	586	30.1 (25.4–34.8)	347	36.3 (30.0–42.6)	239	21.7 (14.6–28.8)
Stage 4/distant	391	19.7 (14.8–24.6)	230	23.2 (18.5–27.9)	161	14.9 (7.8–22.0)
Missing	1260	48.4 (44.7–52.1)	754	62.7 (58.0–67.4)	506	29.5 (23.8–35.2)
**5‐year survival by histological group subtype**						
Squamous carcinoma	5940	71.1 (69.3–72.9)	4546	77.0 (75.2–78.8)	1394	52.7 (48.6–56.8)
Adenocarcinoma	1555	70.1 (66.8–73.4)	1133	81.0 (77.9–84.1)	422	40.0 (36.9–51.1)
Adenosquamous carcinoma	189	59.8 (50.6–69.0)	153	67.1 (57.1–77.1)	36	36.6 (15.5–57.6)
Others/unspecified	666	47.5 (42.2–52.8)	422	64.9 (58.6–71.2)	244	23.1 (16.0–30.2)

Abbreviation: CI: confidence interval

The survival analysis using Cox proportional hazards models yielded a HR of 3.59 (95% CI: 3.40–3.78) comparing elderly women to younger women in the basic model (no adjustment; Table 5). After adjustment for T stage (Model 2), the HR was 2.20 (95% CI: 2.06–2.35). Further adjustment by histological group or treatment did not change the result substantially (Models 3 and 4); however, the HR moved closer toward unity upon adjustment for treatment.

**TABLE 5:** Multivariable Cox proportional hazards model of overall survival in women with cervical cancer in Germany 2001–2015.

	Age group	*N* (events)	HR for overall survival (95% CI)
Model 1 (no adjustment)	**<65**	14,528 (5543)	1.00
	**≥65**		3.59 (3.40–3.78)
Model 2 (adjusted by T stage)	**<65**	12,056 (4025)	1.00
	**≥65**		2.20 (2.06–2.35)
Model 3 (adjusted by T stage, Histological group)	**<65**	12,056 (4025)	1.00
	**≥65**		2.19 (2.06–2.34)
Model 4 (adjusted by T stage, histological group, any treatment)	**<65**	6014 (2004)	1.00
	**≥65**		2.11 (1.93–2.32)

*Note*: Tumor (T) stage includes Stage 1/local, Stage 2/regional, Stage 3/regional, and Stage 4/distant; Histological groups include squamous carcinoma, adenocarcinoma, adenosquamous carcinoma, others/unspecified; any treatment includes surgery, chemotherapy, or radiation therapy.

Abbreviations: CI, confidence interval; HR, hazard ratio.

## 4 DISCUSSION

We provide an in‐depth overview of incidence, treatment and survival of cervical cancer in women in Germany using epidemiological cancer registry data. Overall, elderly women accounted for nearly a third of all cervical cancer cases (27.6%). Hysterectomy correction showed that incidence rates were underestimated and by up to 65% particularly in women aged 70–79. Elderly women were reported to have received surgery or chemotherapy across all cancer stages less often compared to younger women. We observed pronounced differences in relative survival between elderly women and young women with 5‐year relative survival of 46.9% and 76.7%, respectively. The observed cumulative (2001–2015) age‐standardized incidence rate for cervical cancer was 12.5 per 100,000. In line with previous analyses from the USA and Germany,^13^, ^14^ we show that the incidence of cervical cancer is underestimated particularly in elderly women due to a lack of correction accounting for hysterectomy. In the age group 60–69, we observed changes in incidence (32.4%) after hysterectomy correction. Rositch et al. reported changes of 67.3% in women aged 60–64 comparing uncorrected and corrected rates in the US.^13^ These findings need to be considered in the framework of cervical cancer screening and treatment practices.

Internationally, screening is typically offered until the age of 65. However, if incidence estimates in older women are underestimated, a review of screening exit‐age may be warranted. Participation in screening decreases steadily with age and is particularly low among women with low socioeconomic status.^9^, ^12^, ^33^, ^34^ In a population‐based case–control study of cervical cancer in Germany, around 50% of cases aged 60–79 years did not participate in screening in the 10 years prior to diagnosis.^35^ Therefore, a significant proportion of older women have an inadequate screening history. These women could benefit from further screening examinations even after the age of 65. Within the new organized screening system in Germany where women receive information letters up to age 65, women beyond 65 years are not in the focus any more. Recent guidelines from the US suggest that cessation of screening should be determined by previous screening history (e.g. three negative cytology smears or two negative human papillomavirus (HPV) tests within the preceding 10 years).^5^ Extending the exit‐age for screening to 70–74 years could lead to a further 2–3% reduction in cervical cancer incidence.^36^ If women have an inadequate screening history, the results from our study emphasize support for the policy to continue screening until adequate screening is achieved. Modeling studies support this idea, especially for underscreened elderly women.^37^ Furthermore, the personal preferences and additional risk factors of the woman, for example immunosuppression, must be taken into account.

The impact of prophylactic vaccines against high‐risk HPV types associated with the development of CC should also be considered.^38^ HPV vaccines have only been introduced in 2006 in many countries and are recommended for girls aged 9–14 years, and recently also for boys. It is likely that vaccination effects cannot be observed in our analyses with diagnosis years between 2001 and 2015, although some women aged 20–25 in 2015 could have potentially been vaccinated against HPV. However, our trend analysis for the age group 20–29 does not show any decrease in cervical cancer incidence, which might be due to low vaccination coverage at the time. In the future, as younger vaccinated cohorts move into screening eligibility and HPV screening becomes the standard screening test, a re‐evaluation of appropriate screening exit‐age will be necessary for effective screening programs.

An explanation for worse survival among elderly women involves the clinical management (or lack thereof). International results on treatment of cervical cancer in elderly women are in line with our results reporting undertreatment and less aggressive treatment in the elderly compared to younger women.^18^, ^19^ However, due to the low numbers in several subgroups in our analysis, these results need to be interpreted with caution. Sharma et al. analyzed SEER data and reported differences in surgical management between older and younger women.^17^ For localized CC, primary surgery was performed in 82.0% of women <50 years old but only in 54.5% of those 70–79 years of age and 33.2% of women ≥80 years of age. Also, for chemotherapy, another SEER analysis concluded that there was a lack of standard of care for older women (>65 years),^39^ although evidence suggests that any form of treatment is associated with better prognosis compared to no treatment.^19^

Furthermore, most physicians treating advanced CC do not decide objectively about treatment in elderly patients.^40^ Without using frailty screening tools, physicians report adherence to standard care in “fit” elderly patients but administer less intensive treatments to women deemed as “unfit” patients. Further factors, which might contribute to these differences in disease management, might be the presence and severity of comorbidities or older patients refusing aggressive treatments.^41^, ^42^ Undertreatment might explain why our study found lower relative survival rates for elderly cervical cancer patients (≥65 years; 5‐year relative survival: 46.9, 95% CI: 43.8–50.0) compared to younger patients (<65 years; 5‐year relative survival 76.7, 95% CI: 75.1–78.3). A SEER analysis also reported 5‐year cancer‐specific survival rates being lower in elderly patients (59.38%) compared to younger patients (75.02%).^19^ For younger women, 5‐year survival rates were similar to US data, but 5‐year survival rates for older patients <65 years of age were lower in our analyses compared to the US (46.9% vs. 59.38%). Although older women are more likely to be diagnosed with advanced cancer,^20^, ^43^ a greater proportion of older women in our analyses received radiation therapy for T stages 1–2 than women aged <65 years.

Comparing older women to younger women, we also observed decreasing trends in CC incidence for all age groups older than 30 years during the observation period 2001–2015, while incidence increased in the youngest age group 20–29 years of age. Studies conducted in the UK and Norway also found increasing incidence rates for young women, especially for those aged 25–29.^44^, ^45^ However, a US study observed decreasing incidence trends in young women aged 21–23 years and stable trends for women 24–25 years of age.^46^ A study conducted in Canada also reported decreasing trends for cervical cancer incidence in all age groups, including women aged 25–39 years.^47^ This rise in incidence in young women in Europe might be due to low screening uptake in the age group 20–29.^9^ Furthermore, cytology screening performs better in younger women (20–30 years) than older (>50) due to the more visible transformation zone, aiding in collection of sufficient cervical samples. Another reason for the increase could be due to increasing glandular lesions that are detected by the shift in screening tools used (during this time period, guidelines shifted recommendations of cotton swabs towards the use of spatulas and cytobrushes to reach cells from the endocervical canal).^48^ Indeed, an increase in adenocarcinoma was found in young women across several HIC, including the Netherlands^49^, ^50^, ^51^ and highlights greater efforts needed to address inadequate screening of specific age groups.

A strength of this study is the large sample size using data from six cancer registries in Germany, covering approximately 19% of the population. We presented incidence rates uncorrected and corrected for hysterectomy and also incidence trends by age groups. This study provides a detailed overview of cervical cancer epidemiology, particularly in elderly women, including a comprehensive presentation of cancer therapy stratified by age groups. Limitations of our analyses are related to the high proportions of missing values for disease stage or therapy in the cancer registry data. This is due to the limited individual data available from the ZfKD, which relies on data from federal cancer registries. Additionally, the data from the cancer registries did not contain information on screening participation, demographics or comorbidities, which might have influenced decisions on treatment routes and survival outcomes.^52^

According to these analyses, the incidence of CC is underestimated when hysterectomy is not considered in estimations and this underestimation is substantial among older age groups. Furthermore, elderly women with cervical cancer are managed differently and have worse overall survival compared to younger women. Due to the high disease burden in elderly women, screening strategies and treatment recommendations need to be re‐evaluated, improved, and adapted.

## AUTHOR CONTRIBUTIONS

**Sonja Neumeyer:** Data curation (equal); formal analysis (lead); investigation (equal); methodology (equal); visualization (lead); writing – original draft (lead). **Luana Fiengo Tanaka:** Conceptualization (lead); investigation (equal); methodology (lead); project administration (lead); supervision (equal); validation (equal); writing – review and editing (equal). **Linda A. Liang:** Methodology (supporting); validation (equal); visualization (supporting); writing – review and editing (equal). **Stefanie J Klug:** Conceptualization (equal); funding acquisition (equal); investigation (equal); methodology (equal); resources (equal); software (equal); supervision (equal); writing – review and editing (equal).

## FUNDING INFORMATION

No specific funding was disclosed.

## CONFLICT OF INTEREST STATEMENT

The authors made no disclosures.

## ETHICS STATEMENT

This study analyzed the scientific use file (SUF) of the German Centre for Cancer Registry Data (ZfKD). Data collection is mandated and regulated by the Federal Cancer Registry Data Act (BKRG) and secondary data analysis of the anonymous SUF, as used in this study, does not require ethics approval (§3 and §5 BKRG). This study conforms to the Declaration of Helsinki and Good Epidemiological Practices.

## Supporting information


**Data S1:** Supporting Information.

## Data Availability

The data that support the findings of this study are available from German Center for Cancer Registry Data, Robert Koch Institute, Berlin. Restrictions may apply to the availability of these data, which were used for this study.
