# Supplementary material for: Epidemiology of cervical cancer in elderly women: Analysis of incidence, treatment, and survival using German registry data
Source: Cancer Med. 2023 Jul 5;12(16):17284–95. doi: 10.1002/cam4.6318 (PMC10501271; doi:10.1002/cam4.6318)
Supplement: Supplementary file 1 — Data S1: Supporting Information. [file CAM4-12-17284-s001.docx]

**Supplementary Material**

**Epidemiology of Cervical Cancer in Elderly Women: Analysis of Incidence, Therapy and Survival using German Registry Data**

Running title: Cervical cancer in elderly women

Sonja Neumeyer ^a^, Luana Fiengo Tanaka ^a^, Linda A. Liang ^a^, Stefanie J. Klug ^a^

^a^ Chair of Epidemiology, Department of Sport and Health Sciences, Technical University of Munich, Munich, Germany.

**Corresponding author:**

Prof. Dr. Stefanie J. Klug, MPH
Chair of Epidemiology

Department of Sport and Health Sciences

Technical University of Munich

Georg-Brauchle-Ring 56

80992 Munich

Germany

Tel. +49 (0)89-289-24951

Fax +49 (0)89-289-24953
Email: sekretariat.klug@tum.de

**Supplementary Table 1:** Distribution of tumour characteristics of cervical cancer cases according to age groups including Death Certificate only (DCO) cases

|  | |  |  | |  | **Age groups** | | | | | | | | | | |  |  |  | **Age groups** | | |
| --- | --- | --- | --- | --- | --- | --- | --- | --- | --- | --- | --- | --- | --- | --- | --- | --- | --- | --- | --- | --- | --- | --- |
|  | |  |  |  | **Age^1^** | **20-34** | | **35-49** | | **50-64** | | **65-74** | | **75-84** | | **>85** | |  | **< 65** | | **≥ 65** | |
|  | | | **N** | **%** |  | **N** | **%** | **N** | **%** | **N** | **%** | **N** | **%** | **N** | **%** | **N** | **%** |  | **N** | **%** | **N** | **%** |
| **N cases (proportion per age group)** | | | 15363 |  | 55.7 | 1520 | 9.9 | 5067 | 33.0 | 4096 | 26.7 | 2188 | 14.2 | 1713 | 11.2 | 779 | 5.1 |  | 10683 | 69.5 | 4680 | 30.5 |
|  |  | |  |  |  |  |  |  |  |  |  |  |  |  |  |  |  |  |  |  |  |  |
| **Histology (N (%))** | | |  |  |  |  |  |  |  |  |  |  |  |  |  |  |  |  |  |  |  |  |
|  | **Squamous carcinoma** | | 10597 | 69.0 | 53.8 | 1152 | 75.8 | 3758 | 74.2 | 2971 | 72.5 | 1447 | 66.1 | 944 | 55.1 | 325 | 41.7 |  | 7881 | 73.8 | 2716 | 58.0 |
|  | **Adenocarcinoma** | | 2577 | 16.8 | 57.6 | 252 | 16.6 | 875 | 17.3 | 663 | 16.2 | 382 | 17.5 | 302 | 17.6 | 103 | 13.2 |  | 1790 | 16.8 | 787 | 16.8 |
|  | **Adenosquamous carcinoma** | | 342 | 2.2 | 52.5 | 31 | 2.0 | 146 | 2.9 | 89 | 2.2 | 48 | 2.2 | 25 | 1.5 | 3 | 0.4 |  | 266 | 2.5 | 76 | 1.6 |
|  | **Others / unspecified** | | 1847 | 12.0 | 68.0 | 85 | 5.6 | 288 | 5.7 | 373 | 9.1 | 311 | 14.2 | 442 | 25.8 | 348 | 44.7 |  | 746 | 7.0 | 1101 | 23.5 |
|  | | |  |  |  |  |  |  |  |  |  |  |  |  |  |  |  |  |  |  |  |  |
| **Stage (N (%))** | | |  |  |  |  |  |  |  |  |  |  |  |  |  |  |  |  |  |  |  |  |
|  | **Stage 1 / local** | | 7151 | 46.5 |  | 1194 | 78.6 | 3276 | 64.7 | 1625 | 39.7 | 653 | 29.8 | 331 | 19.3 | 72 | 9.2 |  | 6095 | 57.1 | 1056 | 22.6 |
|  | **Stage 2 / regional** | | 2803 | 18.2 |  | 134 | 8.8 | 784 | 15.5 | 961 | 23.5 | 521 | 23.8 | 314 | 18.3 | 89 | 11.4 |  | 1879 | 17.6 | 924 | 19.7 |
|  | **Stage 3 / regional** | | 1252 | 8.1 |  | 19 | 1.3 | 266 | 5.2 | 425 | 10.4 | 237 | 10.8 | 227 | 13.3 | 78 | 10.0 |  | 710 | 6.6 | 542 | 11.6 |
|  | **Stage 4 / distant** | | 858 | 5.6 |  | 21 | 1.4 | 162 | 3.2 | 308 | 7.5 | 187 | 8.5 | 133 | 7.8 | 47 | 6.0 |  | 491 | 4.6 | 367 | 7.8 |
|  | **missing** | | 3299 | 21.5 |  | 152 | 10.0 | 579 | 11.4 | 777 | 19.0 | 590 | 27.1 | 708 | 41.3 | 493 | 63.3 |  | 1508 | 14.1 | 1791 | 38.3 |
|  | | |  |  |  |  |  |  |  |  |  |  |  |  |  |  |  |  |  |  |  |  |
| **Grading (N (%))** | | |  |  |  |  |  |  |  |  |  |  |  |  |  |  |  |  |  |  |  |  |
|  | **Well differentiated** | | 1051 | 6.8 |  | 198 | 13.0 | 466 | 9.2 | 227 | 5.5 | 87 | 4.0 | 58 | 3.4 | 15 | 1.9 |  | 891 | 8.3 | 160 | 3.4 |
|  | **Moderately differentiated** | | 6284 | 40.9 |  | 645 | 42.4 | 2280 | 45.0 | 1770 | 43.2 | 865 | 39.5 | 535 | 31.2 | 189 | 24.3 |  | 4695 | 43.9 | 1589 | 34.0 |
|  | **Poorly differentiated** | | 5061 | 32.9 |  | 398 | 26.2 | 1651 | 32.6 | 1497 | 36.5 | 780 | 35.6 | 561 | 32.7 | 174 | 22.3 |  | 3546 | 33.2 | 1515 | 32.4 |
|  | **Undifferentiated** | | 120 | 0.8 |  | 9 | 0.6 | 23 | 0.5 | 30 | 0.7 | 28 | 1.3 | 21 | 1.2 | 9 | 1.2 |  | 62 | 0.6 | 58 | 1.2 |
|  | **missing** | | 2847 | 0.8 |  | 270 | 17.8 | 647 | 12.8 | 572 | 14.0 | 428 | 19.6 | 538 | 31.4 | 392 | 50.3 |  | 1489 | 13.9 | 1358 | 29.0 |
|  | | |  | |  |  | |  | |  |  |  |  |  |  |  |  |  |  |  |  |  |

^1^Mean age at diagnosis

**Supplementary Table 2:** Numbers of cases and age-adjusted incidence rates of cervical cancer per 100.000 women by year of diagnosis 2001-2015, uncorrected and corrected for hysterectomy prevalence among women in Germany compared to reported rates for Germany by the Robert Koch Institute (RKI)

|  | |  | **2001** | **2002** | | | **2003** | **2004** | **2005** | **2006** | **2007** | **2008** | **2009** | **2010** | | **2011** | **2012** | | **2013** | | **2014** | | **2015** |
| --- | --- | --- | --- | --- | --- | --- | --- | --- | --- | --- | --- | --- | --- | --- | --- | --- | --- | --- | --- | --- | --- | --- | --- |
| **New cases in this analysis (n)** | |  | 1096 | | 1037 | 1010 | | 987 | 914 | 1010 | 987 | 979 | 1007 | | 927 | 930 | | 918 | | 950 | | 915 | 861 |
| **New cases in total Germany according to RKI^b^ (n)** | |  | 5821 | | 5444 | 5180 | | 5299 | 4729 | 5133 | 5115 | 5138 | 5107 | | 4933 | 4773 | | 4817 | | 4637 | | 4654 | 4489 |
| **Crude incidence rate in total Germany according to RKI ^b^** | |  | 13.8 | | 13.0 | 14.2 | | 12.7 | 11.3 | 12.3 | 12.2 | 12.3 | 12.2 | | 11.8 | 11.7 | | 11.6 | | 11.3 | | 11.1 | 10.8 |
| **Age-standardized rate in total Germany (European standard) according to RKI (not corrected for hysterectomy)^b^** | |  | 11.5 | | 10.8 | 10.3 | | 10.4 | 9.5 | 10.2 | 10.2 | 10.1 | 10.1 | | 9.9 | 9.5 | | 9.5 | | 9.4 | | 9.3 | 9.1 |
| **Crude incidence rate in this analysis** | |  | 14.8 | | 14.0 | 13.7 | | 13.4 | 12.4 | 13.8 | 13.5 | 13.4 | 13.9 | | 12.8 | 13.1 | | 12.8 | | 13.3 | | 12.8 | 12.0 |
|  | |  |  | |  |  | |  |  |  |  |  |  | |  |  | |  | |  | |  |  |
| **Age-standardized incidence rate (old European standard)^c^** | |  |  | |  |  | |  |  |  |  |  |  | |  |  | |  | |  | |  |  |
| Hysterectomy | Not corrected |  | 13.9 | | 13.1 | 12.7 | | 12.4 | 11.8 | 13.0 | 12.6 | 12.6 | 13.1 | | 12.0 | 12.0 | | 12.0 | | 12.5 | | 12.1 | 11.3 |
|  | Corrected |  | 17.4 | | 16.3 | 15.9 | | 15.6 | 14.8 | 16.1 | 15.8 | 15.7 | 16.2 | | 15.0 | 15.0 | | 14.9 | | 15.4 | | 15.0 | 14.0 |
| **Age-standardized incidence rate (US standard)^d^** | |  |  | |  |  | |  |  |  |  |  |  | |  |  | |  | |  | |  |  |
| Hysterectomy | Not corrected |  | 13.6 | | 12.9 | 12.4 | | 12.2 | 11.4 | 12.7 | 12.3 | 12.3 | 12.8 | | 11.7 | 11.6 | | 11.7 | | 12.2 | | 11.7 | 10.9 |
|  | Corrected |  | 16.9 | | 15.9 | 15.4 | | 15.2 | 14.2 | 15.6 | 15.3 | 15.2 | 15.7 | | 14.5 | 14.5 | | 14.5 | | 15.0 | | 14.4 | 13.5 |
| **Age standardized incidence rate (World standard)^e^** | |  |  | |  |  | |  |  |  |  |  |  | |  |  | |  | |  | |  |  |
| Hysterectomy | Not corrected |  | 11.6 | | 11.0 | 10.6 | | 10.3 | 9.9 | 10.9 | 10.6 | 10.6 | 11.1 | | 10.1 | 10.0 | | 10.2 | | 10.5 | | 10.2 | 9.6 |
|  | Corrected |  | 14.2 | | 13.3 | 13.0 | | 12.6 | 12.1 | 13.2 | 12.9 | 12.9 | 13.4 | | 12.3 | 12.2 | | 12.3 | | 12.8 | | 12.4 | 11.6 |
|  | |  |  | |  |  | |  |  |  |  |  |  | |  |  | |  | |  | |  |  |

^a^ Incidence rates corrected and uncorrected for hysterectomy prevalence with population at risk reduced using hysterectomy prevalences by Prütz et al. (2013)^1^

**^b^** Number of cervical cancer cases and incidence by year for Germany were extracted from the database of the German Center for Cancer Registry data (ZfKD) ^2^

^c^Age-adjusted using the old European standard population^3^

^d^Age-adjusted using the standard 2000 US census population^4^

^e^Age-adjusted using the world standard population^5^

Abbreviations: RKI, Robert Koch Institute

**Supplementary Table 3:** Trends in incidence rates of cervical cancer per 100 000 women (not corrected for hysterectomy) by age group and histological type (adenocarcinoma n=2 417, squamous carcinoma n=10 439, adenoquamous carcinoma n=342)

|  | |  | **2001** | **2002** | | **2003** | **2004** | | **2005** | | **2006** | **2007** | | **2008** | | **2009** | **2010** | | **2011** | | **2012** | | **2013** | | **2014** | | **2015** | |
| --- | --- | --- | --- | --- | --- | --- | --- | --- | --- | --- | --- | --- | --- | --- | --- | --- | --- | --- | --- | --- | --- | --- | --- | --- | --- | --- | --- | --- |
| **20-29 years** | |  |  |  |  | | |  |  |  | | |  | |  |  | |  | |  | |  | |  | |  | |  |
| **Incidence rate** | |  |  |  |  | | |  |  |  | | |  | |  |  | |  | |  | |  | |  | |  | |  |
|  | **All cases** |  | 4.01 | 1.94 | 2.82 | | | 3.67 | 2.86 | 3.9 | | | 3.89 | | 4.00 | 4.44 | | 3.69 | | 3.71 | | 4.67 | | 4.39 | | 4.33 | | 5.66 |
|  | **Squamous carcinoma** |  | 2.64 | 1.71 | 2.14 | | | 2.68 | 2.20 | 2.61 | | | 3.24 | | 3.13 | 3.46 | | 2.71 | | 2.58 | | 4.10 | | 3.35 | | 3.86 | | 3.53 |
|  | **Adenocarcinoma** |  | 0.80 | 0.11 | 0.34 | | | 0.89 | 0.44 | 0.76 | | | 0.43 | | 0.54 | 0.65 | | 0.54 | | 1.01 | | 0.23 | | 0.69 | | 0.35 | | 1.06 |
|  | **Adenosquamous carcinoma** |  | 0.00 | 0.00 | 0.23 | | | 0.00 | 0.00 | 0.00 | | | 0.00 | | 0.22 | 0.00 | | 0.33 | | 0.00 | | 0.11 | | 0.00 | | 0.00 | | 0.12 |
| **30-39 years** | |  |  |  |  | | |  |  |  | | |  | |  |  | |  | |  | |  | |  | |  | |  |
| **Incidence rate** | |  |  |  |  | | |  |  |  | | |  | |  |  | |  | |  | |  | |  | |  | |  |
|  | **All cases** |  | 18.84 | 20.49 | 17.06 | | | 17.25 | 15.52 | 18.94 | | | 15.86 | | 16.10 | 16.23 | | 15.52 | | 15.61 | | 15.95 | | 16.29 | | 16.77 | | 15.06 |
|  | **Squamous carcinoma** |  | 13.60 | 15.32 | 13.70 | | | 13.54 | 11.45 | 14.28 | | | 11.98 | | 11.75 | 10.97 | | 10.54 | | 12.11 | | 11.67 | | 11.73 | | 11.74 | | 10.70 |
|  | **Adenocarcinoma** |  | 2.87 | 2.54 | 1.77 | | | 2.13 | 2.52 | 3.44 | | | 2.31 | | 2.83 | 3.36 | | 3.17 | | 2.45 | | 3.24 | | 3.19 | | 3.59 | | 2.84 |
|  | **Adenosquamous carcinoma** |  | 0.57 | 0.59 | 0.18 | | | 0.65 | 0.39 | 0.30 | | | 0.42 | | 0.54 | 0.22 | | 0.45 | | 0.35 | | 0.12 | | 0.22 | | 0.34 | | 0.44 |
| **40-49 years** | |  |  |  |  | | |  |  |  | | |  | |  |  | |  | |  | |  | |  | |  | |  |
| **Incidence rate** | |  |  |  |  | | |  |  |  | | |  | |  |  | |  | |  | |  | |  | |  | |  |
|  | **All cases** |  | 21.18 | 20.61 | 20.17 | | | 17.51 | 19.22 | 19.74 | | | 20.82 | | 21.46 | 23.50 | | 18.62 | | 17.38 | | 19.07 | | 21.42 | | 18.62 | | 16.83 |
|  | **Squamous carcinoma** |  | 15.82 | 15.50 | 15.65 | | | 13.46 | 14.97 | 15.46 | | | 15.14 | | 16.09 | 17.69 | | 12.82 | | 12.28 | | 12.95 | | 14.76 | | 13.83 | | 11.74 |
|  | **Adenocarcinoma** |  | 2.72 | 2.09 | 2.55 | | | 2.27 | 2.56 | 2.62 | | | 2.84 | | 3.63 | 4.46 | | 3.56 | | 3.17 | | 3.88 | | 4.77 | | 2.82 | | 3.13 |
|  | **Adenosquamous carcinoma** |  | 0.68 | 0.92 | 0.41 | | | 0.65 | 0.32 | 0.32 | | | 0.87 | | 0.63 | 0.32 | | 0.73 | | 0.58 | | 0.60 | | 0.81 | | 0.66 | | 0.78 |
| **50-59 years** | |  |  |  |  | | |  |  |  | | |  | |  |  | |  | |  | |  | |  | |  | |  |
| **Squamous carcinoma** | |  |  |  |  | | |  |  |  | | |  | |  |  | |  | |  | |  | |  | |  | |  |
|  | **All cases** |  | 19.30 | 17.06 | 16.69 | | | 18.31 | 17.32 | 18.21 | | | 18.60 | | 16.97 | 17.43 | | 18.00 | | 18.40 | | 16.47 | | 16.16 | | 16.45 | | 14.90 |
|  | **Squamous carcinoma** |  | 15.70 | 12.04 | 12.23 | | | 14.22 | 12.89 | 13.66 | | | 13.70 | | 12.98 | 13.68 | | 12.09 | | 13.77 | | 13.46 | | 11.45 | | 11.89 | | 10.66 |
|  | **Adenocarcinoma** |  | 1.91 | 3.04 | 2.18 | | | 1.64 | 2.26 | 2.75 | | | 2.59 | | 2.36 | 2.41 | | 3.97 | | 3.05 | | 2.06 | | 2.78 | | 2.98 | | 2.93 |
|  | **Adenosquamous carcinoma** |  | 0.42 | 0.42 | 0.21 | | | 0.20 | 0.49 | 0.47 | | | 0.64 | | 0.55 | 0.09 | | 0.35 | | 0.44 | | 0.17 | | 0.67 | | 0.50 | | 0.24 |
| **60-69 years** | |  |  |  |  | | |  |  |  | | |  | |  |  | |  | |  | |  | |  | |  | |  |
| **Squamous carcinoma** | |  |  |  |  | | |  |  |  | | |  | |  |  | |  | |  | |  | |  | |  | |  |
|  | **All cases** |  | 16.72 | 16.00 | 17.63 | | | 13.76 | 15.14 | 15.14 | | | 14.52 | | 15.16 | 15.32 | | 14.94 | | 14.61 | | 13.78 | | 14.50 | | 15.90 | | 14.34 |
|  | **Squamous carcinoma** |  | 12.78 | 12.84 | 13.41 | | | 10.35 | 9.58 | 11.26 | | | 9.92 | | 10.84 | 11.65 | | 10.11 | | 10.39 | | 9.04 | | 11.01 | | 11.71 | | 8.87 |
|  | **Adenocarcinoma** |  | 2.07 | 1.58 | 2.39 | | | 1.76 | 2.78 | 1.79 | | | 2.66 | | 2.95 | 2.27 | | 3.41 | | 3.02 | | 3.09 | | 1.96 | | 3.44 | | 3.51 |
|  | **Adenosquamous carcinoma** |  | 0.38 | 0.28 | 0.28 | | | 0.46 | 0.29 | 0.20 | | | 0.61 | | 0.32 | 0.43 | | 0.22 | | 0.22 | | 0.55 | | 0.11 | | 0.43 | | 0.10 |
| **70-79 years** | |  |  |  |  | | |  |  |  | | |  | |  |  | |  | |  | |  | |  | |  | |  |
| **Squamous carcinoma** | |  |  |  |  | | |  |  |  | | |  | |  |  | |  | |  | |  | |  | |  | |  |
|  | **All cases** |  | 18.93 | 15.38 | 14.81 | | | 17.62 | 12.74 | 14.08 | | | 15.07 | | 13.47 | 14.25 | | 11.68 | | 13.10 | | 12.86 | | 13.85 | | 12.12 | | 12.80 |
|  | **Squamous carcinoma** |  | 13.98 | 9.82 | 8.78 | | | 13.22 | 8.92 | 9.22 | | | 9.36 | | 9.22 | 11.29 | | 6.61 | | 8.80 | | 8.40 | | 8.71 | | 6.11 | | 8.86 |
|  | **Adenocarcinoma** |  | 2.29 | 3.49 | 2.75 | | | 2.33 | 1.53 | 2.62 | | | 4.25 | | 1.77 | 2.05 | | 2.64 | | 2.68 | | 2.87 | | 2.62 | | 3.90 | | 2.74 |
|  | **Adenosquamous carcinoma** |  | 0.25 | 0.26 | 0.66 | | | 0.39 | 0.38 | 0.37 | | | 0.24 | | 0.12 | 0.11 | | 0.44 | | 0.12 | | 0.12 | | 0.73 | | 0.42 | | 0.11 |
|  |  |  |  |  |  | | |  |  |  | | |  | |  |  | |  | |  | |  | |  | |  | |  |

**Supplementary Table 4:** Distribution of therapy uptake of cervical cancer cases according to age and tumor stage from 2001-2015 in Germany including death certificate only (DCO) cases

|  | | | |  | |  | | **Age groups** | | | | | | | | | | |  |  | **Age groups** | | | |
| --- | --- | --- | --- | --- | --- | --- | --- | --- | --- | --- | --- | --- | --- | --- | --- | --- | --- | --- | --- | --- | --- | --- | --- | --- |
|  | | | |  |  |  | **20-34** | | | **35-49** | | **50-64** | | **65-74** | | **75-84** | | **>85** | |  | **< 65** | | **≥ 65** | |
|  | | | | **N** | **%** | **N** | | | **%** | **N** | **%** | **N** | **%** | **N** | **%** | **N** | **%** | **N** | **%** |  | **N** | **%** | **N** | **%** |
| **Any treatment** | | | |  |  |  | | |  |  |  |  |  |  |  |  |  |  |  |  |  |  |  |  |
|  | | **yes** | | 6182 | 40.2 | 660 | | | 43.4 | 2239 | 44.2 | 1688 | 42.4 | 928 | 42.4 | 528 | 30.8 | 139 | 17.8 |  | 4587 | 42.9 | 1595 | 34.1 |
|  | | **no** | | 750 | 4.9 | 23 | | | 1.5 | 88 | 1.7 | 144 | 5.7 | 124 | 5.7 | 207 | 12.1 | 164 | 21.1 |  | 255 | 2.4 | 495 | 10.6 |
|  | | **missing** | | 8431 | 54.9 | 837 | | | 55.1 | 2740 | 54.1 | 2264 | 51.9 | 1136 | 51.9 | 978 | 57.1 | 476 | 61.1 |  | 5841 | 54.7 | 2590 | 55.3 |
| **Treatment (N (%))** | | | |  |  |  | | |  |  |  |  |  |  |  |  |  |  |  |  |  |  |  |  |
| **Surgery** | | | |  |  |  | | |  |  |  |  |  |  |  |  |  |  |  |  |  |  |  |  |
| **Stage 1** | | | |  |  |  | | |  |  |  |  |  |  |  |  |  |  |  |  |  |  |  |  |
|  | **Yes** | | | 3308 | 46.3 | 563 | | | 47.2 | 1530 | 46.7 | 734 | 45.2 | 319 | 48.9 | 140 | 42.3 | 22 | 30.6 |  | 2827 | 46.4 | 481 | 45.5 |
|  | **no** | | | 145 | 2.0 | 17 | | | 1.4 | 43 | 1.3 | 30 | 1.8 | 25 | 3.8 | 21 | 6.3 | 9 | 12.5 |  | 90 | 1.5 | 55 | 5.2 |
|  | **missing** | | | 3698 | 51.7 | 614 | | | 51.4 | 1703 | 52.0 | 861 | 53.0 | 309 | 47.3 | 170 | 51.4 | 41 | 56.9 |  | 3178 | 52.1 | 520 | 49.2 |
| **Stage 2** | | | |  |  |  | | |  |  |  |  |  |  |  |  |  |  |  |  |  |  |  |  |
|  | **Yes** | | | 1007 | 35.9 | 51 | | | 38.1 | 305 | 38.9 | 341 | 35.5 | 209 | 40.1 | 83 | 26.4 | 18 | 20.2 |  | 697 | 37.1 | 310 | 33.5 |
|  | **no** | | | 340 | 12.1 | 3 | | | 2.2 | 55 | 7.0 | 111 | 11.6 | 77 | 14.8 | 65 | 20.7 | 29 | 32.6 |  | 169 | 9.0 | 171 | 18.5 |
|  | **missing** | | | 1456 | 51.9 | 80 | | | 59.7 | 424 | 54.1 | 509 | 53.0 | 235 | 45.1 | 166 | 52.9 | 42 | 47.2 |  | 1013 | 53.9 | 443 | 47.9 |
| **Stage 3** | | | |  |  |  | | |  |  |  |  |  |  |  |  |  |  |  |  |  |  |  |  |
|  | **Yes** | | | 173 | 13.8 | 3 | | | 15.8 | 51 | 19.2 | 56 | 13.2 | 30 | 12.7 | 30 | 13.2 | 3 | 3.8 |  | 110 | 15.5 | 63 | 11.6 |
|  | **no** | | | 497 | 39.7 | 3 | | | 15.8 | 80 | 30.1 | 168 | 39.5 | 109 | 46.0 | 103 | 45.4 | 34 | 43.6 |  | 251 | 35.4 | 246 | 45.4 |
|  | **missing** | | | 582 | 46.5 | 13 | | | 68.4 | 135 | 50.8 | 201 | 47.3 | 98 | 41.4 | 94 | 41.4 | 41 | 52.6 |  | 349 | 49.2 | 233 | 43.0 |
| **Stage 4** | | | |  |  |  | | |  |  |  |  |  |  |  |  |  |  |  |  |  |  |  |  |
|  | **Yes** | | | 142 | 16.6 | 8 | | | 38.1 | 35 | 21.6 | 45 | 14.6 | 34 | 18.2 | 17 | 12.8 | 3 | 6.4 |  | 88 | 17.9 | 54 | 14.7 |
|  | **no** | | | 369 | 43.0 | 4 | | | 19.0 | 58 | 35.8 | 137 | 44.5 | 77 | 41.2 | 70 | 52.6 | 23 | 48.9 |  | 199 | 40.5 | 170 | 46.3 |
|  | **missing** | | | 347 | 40.4 | 9 | | | 42.9 | 69 | 42.6 | 126 | 40.9 | 76 | 40.6 | 46 | 34.6 | 21 | 44.7 |  | 204 | 41.5 | 143 | 39.0 |
|  |  | | |  |  |  | | |  |  |  |  |  |  |  |  |  |  |  |  |  |  |  |  |
| **Chemotherapy** | | | |  |  |  | | |  |  |  |  |  |  |  |  |  |  |  |  |  |  |  |  |
| **Stage 1** | | |  | | |  | | |  |  |  |  |  |  |  |  |  |  |  |  |  |  |  |  |
|  | **Yes** | | | 538 | 7.5 | 79 | | | 6.6 | 268 | 8.2 | 139 | 8.6 | 46 | 7.0 | 6 | 1.8 | 0 | 0 |  | 486 | 8.0 | 52 | 4.9 |
|  | **no** | | | 2661 | 37.2 | 472 | | | 39.5 | 1178 | 36.0 | 564 | 34.7 | 276 | 42.3 | 143 | 43.2 | 28 | 38.9 |  | 2214 | 36.3 | 447 | 42.3 |
|  | **missing** | | | 3952 | 55.3 | 643 | | | 53.9 | 1830 | 55.9 | 922 | 56.7 | 331 | 50.7 | 182 | 55.0 | 44 | 61.1 |  | 3395 | 55.7 | 557 | 52.7 |
| **Stage 2** | | |  | | |  | | |  |  |  |  |  |  |  |  |  |  |  |  |  |  |  |  |
|  | **Yes** | | | 654 | 23.3 | 36 | | | 26.9 | 218 | 27.8 | 269 | 28.0 | 112 | 21.5 | 19 | 6.1 | 0 | 0 |  | 523 | 27.8 | 131 | 14.2 |
|  | **no** | | | 617 | 22.0 | 18 | | | 13.4 | 119 | 15.2 | 156 | 16.2 | 158 | 30.3 | 122 | 38.9 | 44 | 49.4 |  | 293 | 15.6 | 324 | 35.1 |
|  | **missing** | | | 1532 | 54.7 | 80 | | | 59.7 | 447 | 57.0 | 536 | 55.8 | 251 | 48.2 | 173 | 55.1 | 45 | 50.6 |  | 1063 | 56.6 | 469 | 50.8 |
| **Stage 3** | | |  | | |  | | |  |  |  |  |  |  |  |  |  |  |  |  |  |  |  |  |
|  | **Yes** | | | 329 | 26.3 | 3 | | | 15.8 | 101 | 38.0 | 146 | 34.4 | 63 | 26.6 | 14 | 6.2 | 2 | 2.6 |  | 250 | 35.2 | 79 | 14.6 |
|  | **no** | | | 344 | 27.5 | 3 | | | 15.8 | 29 | 10.9 | 80 | 18.8 | 75 | 31.6 | 121 | 53.3 | 36 | 46.2 |  | 112 | 15.8 | 232 | 42.8 |
|  | **missing** | | | 579 | 46.2 | 13 | | | 68.4 | 136 | 51.1 | 199 | 46.8 | 99 | 41.8 | 92 | 40.5 | 40 | 51.3 |  | 348 | 49.0 | 231 | 42.6 |
| **Stage 4** | | |  | | |  | | |  |  |  |  |  |  |  |  |  |  |  |  |  |  |  |  |
|  | **Yes** | | | 9879 | 16.0 | 11 | | | 52.4 | 63 | 38.9 | 95 | 30.8 | 38 | 20.3 | 10 | 7.5 | 0 | 0 |  | 169 | 34.4 | 48 | 13.1 |
|  | **no** | | | 18410 | 29.7 | 2 | | | 9.5 | 34 | 21.0 | 86 | 27.9 | 74 | 39.6 | 77 | 57.9 | 26 | 55.3 |  | 122 | 24.8 | 177 | 48.2 |
|  | **missing** | | | 33645 | 54.3 | 8 | | | 38.1 | 65 | 40.1 | 127 | 41.2 | 75 | 40.1 | 46 | 34.6 | 21 | 44.7 |  | 200 | 40.7 | 142 | 38.7 |
|  |  | | |  |  |  | | |  |  |  |  |  |  |  |  |  |  |  |  |  |  |  |  |
| **Radiation** | | | |  |  |  | | |  |  |  |  |  |  |  |  |  |  |  |  |  |  |  |  |
| **Stage 1** | | | |  |  |  | | |  |  |  |  |  |  |  |  |  |  |  |  |  |  |  |  |
|  | **yes** | | | 917 | 12.8 | 95 | | | 8.0 | 379 | 11.6 | 248 | 15.3 | 119 | 18.2 | 68 | 20.5 | 8 | 11.1 |  | 722 | 11.8 | 195 | 18.5 |
|  | **No** | | | 2295 | 32.1 | 457 | | | 38.3 | 1069 | 32.6 | 459 | 28.2 | 206 | 31.5 | 82 | 24.8 | 22 | 30.6 |  | 1985 | 32.6 | 310 | 29.4 |
|  | **missing** | | | 3939 | 55.1 | 642 | | | 53.8 | 1828 | 55.8 | 918 | 56.5 | 328 | 50.2 | 181 | 54.7 | 42 | 58.3 |  | 3388 | 55.6 | 551 | 52.2 |
| **Stage 2** | | |  | | |  | | |  |  |  |  |  |  |  |  |  |  |  |  |  |  |  |  |
|  | **yes** | | | 938 | 33.5 | 38 | | | 28.4 | 252 | 32.1 | 321 | 33.4 | 209 | 40.1 | 95 | 30.3 | 23 | 25.8 |  | 611 | 32.5 | 327 | 35.4 |
|  | **No** | | | 344 | 12.3 | 16 | | | 11.9 | 87 |  | 106 | 11.0 | 63 | 12.1 | 50 | 15.9 | 22 | 24.7 |  | 209 | 11.1 | 135 | 14.6 |
|  | **missing** | | | 1521 | 54.3 | 80 | | | 59.7 | 445 | 56.8 | 534 | 55.6 | 249 | 47.8 | 169 | 53.8 | 44 | 49.4 |  | 1059 | 56.4 | 462 | 50.0 |
| **Stage 3** | | |  | | |  | | |  |  |  |  |  |  |  |  |  |  |  |  |  |  |  |  |
|  | **yes** | | | 535 | 42.7 | 4 | | | 21.1 | 113 | 42.5 | 183 | 43.1 | 103 | 43.5 | 102 | 44.9 | 30 | 38.5 |  | 300 | 42.3 | 235 | 43.4 |
|  | **No** | | | 141 | 11.3 | 2 | | | 10.5 | 18 | 6.8 | 42 | 9.9 | 35 | 14.8 | 33 | 14.5 | 11 | 14.1 |  | 62 | 8.7 | 79 | 14.6 |
|  | **missing** | | | 576 | 46.0 | 13 | | | 68.4 | 135 | 50.8 | 200 | 47.1 | 99 | 41.8 | 92 | 40.5 | 37 | 47.4 |  | 348 | 49.0 | 228 | 42.1 |
|  |  | | |  |  |  | | |  |  |  |  |  |  |  |  |  |  |  |  |  |  |  |  |
| **Stage 4** | | |  | | |  | | |  |  |  |  |  |  |  |  |  |  |  |  |  |  |  |  |
|  | **yes** | | | 351 | 40.9 | 11 | | | 52.4 | 73 | 45.1 | 136 | 44.2 | 66 | 35.3 | 54 | 40.6 | 11 | 23.4 |  | 220 | 44.8 | 131 | 35.7 |
|  | **No** | | | 168 | 19.6 | 2 | | | 9.5 | 25 | 15.4 | 47 | 15.3 | 46 | 24.6 | 33 | 24.8 | 15 | 31.9 |  | 74 | 15.1 | 94 | 25.6 |
|  | **missing** | | | 339 | 39.5 | 8 | | | 38.1 | 64 | 38.1 | 125 | 40.6 | 75 | 40.1 | 46 | 34.6 | 21 | 44.7 |  | 197 | 40.1 | 142 | 38.7 |


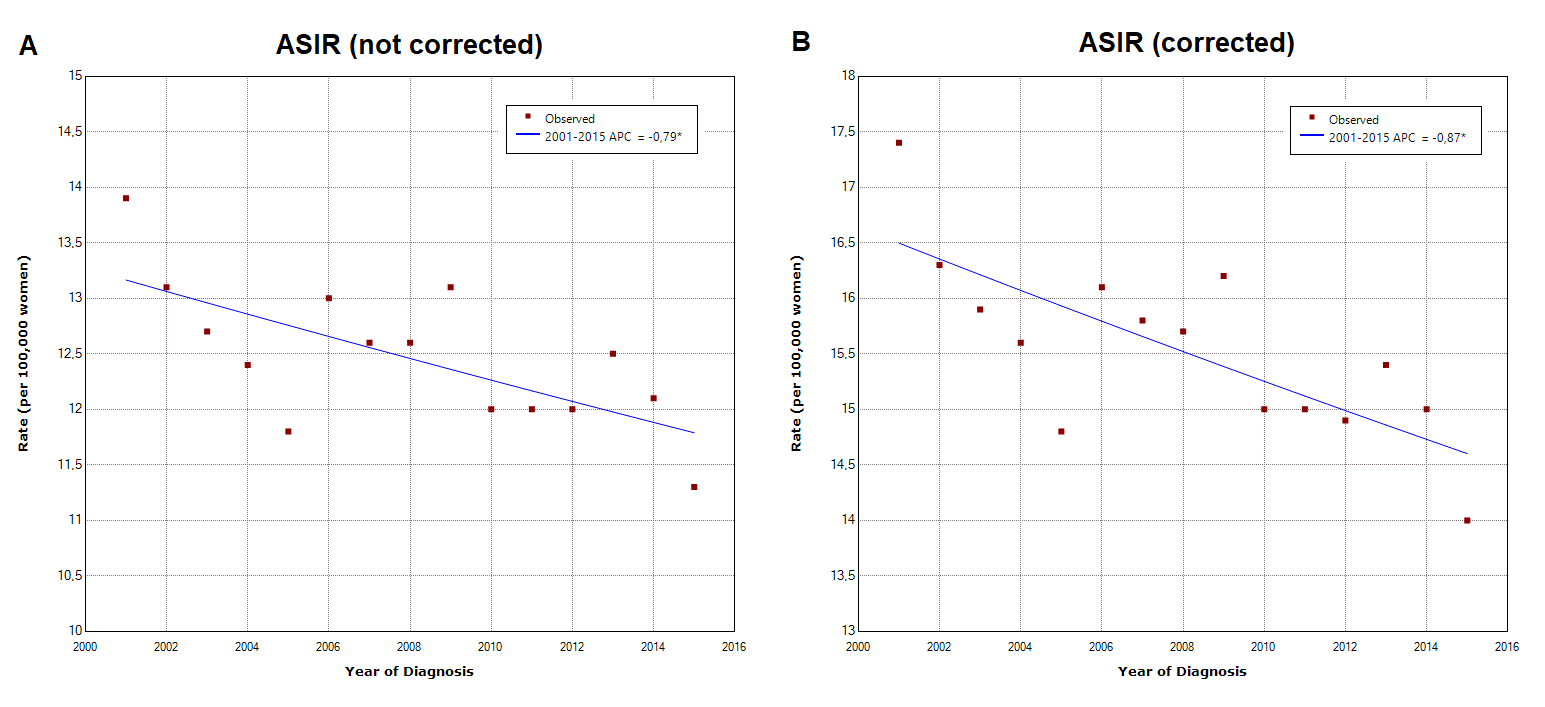


**Supplementary Figure 1:** Trends in age-standardized incidence rates (ASIR) of cervical cancer between 2001 and 2016; A shows rates not corrected for hysterectomy, B shows rates corrected for hysterectomy. * Indicates that the annual percent change (APC) is significantly different from zero at p-value<0.05. Final selected models for all age groups: 0 joinpoints.

**A Age 20-29 years B Age 30-39 years**


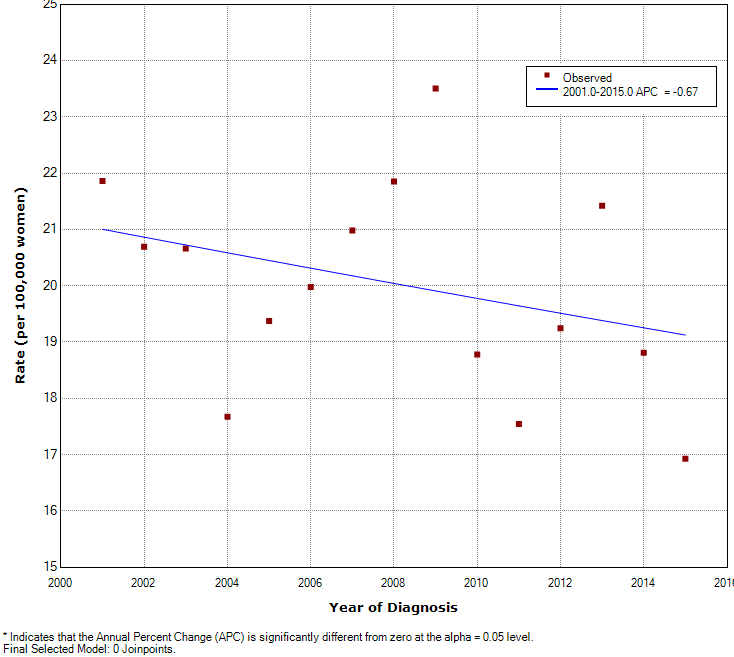

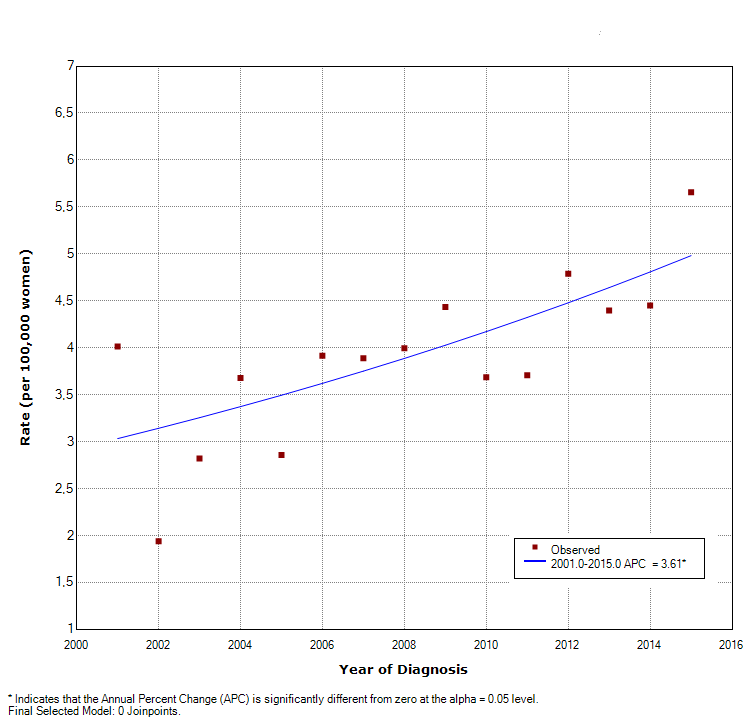

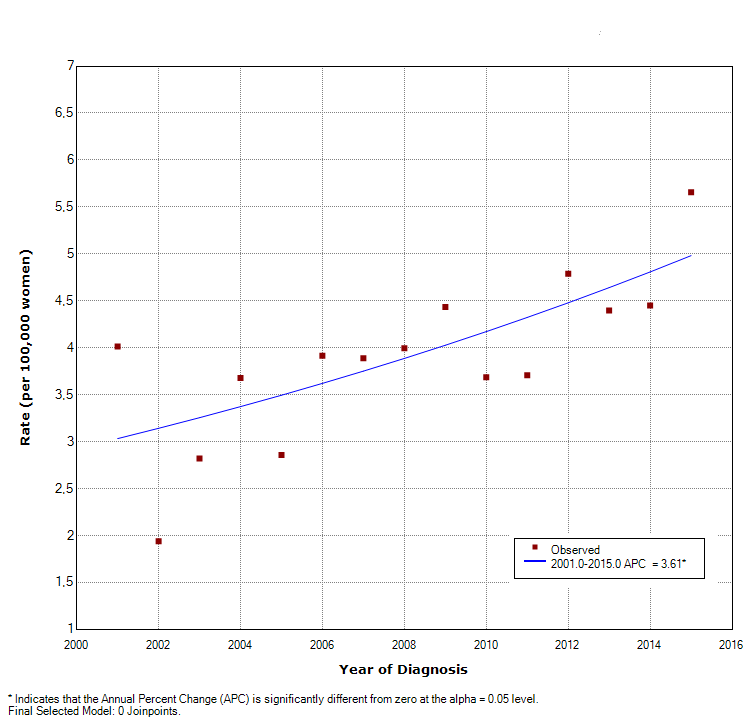

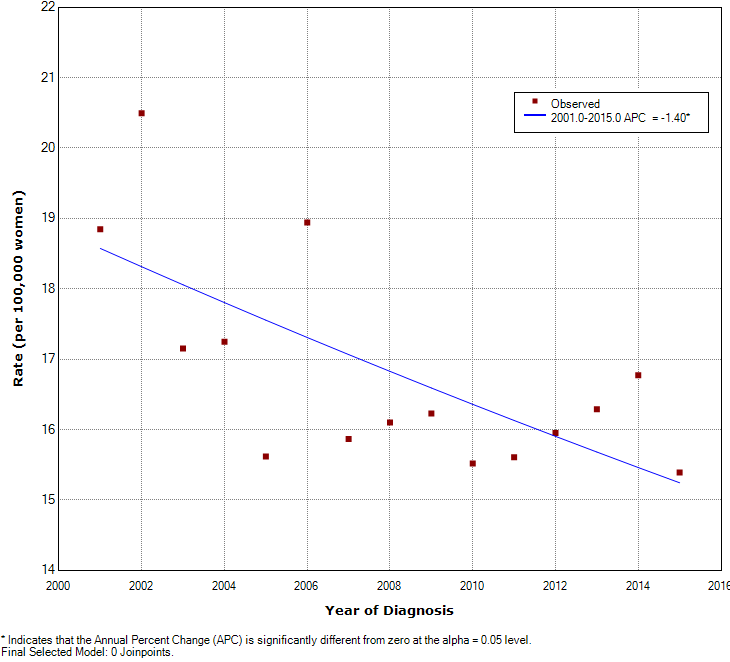

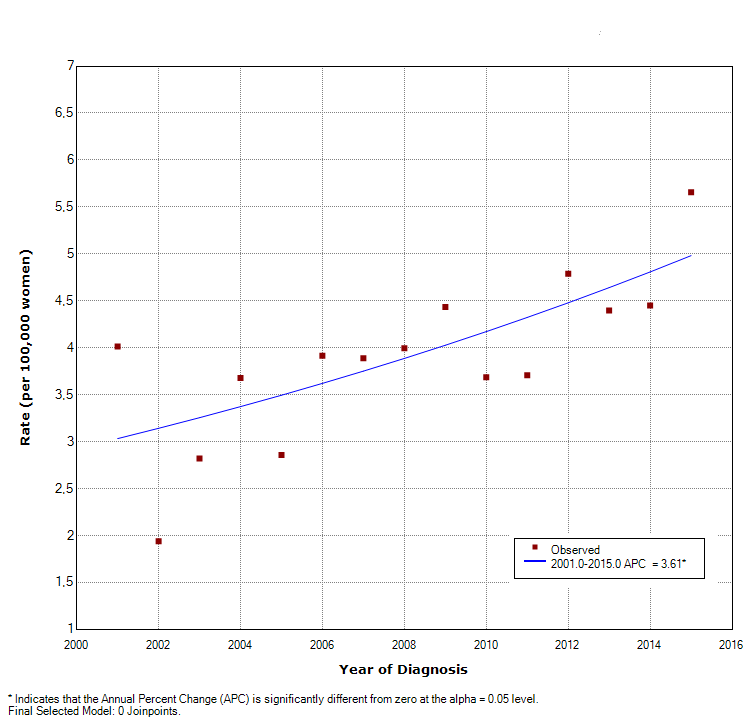

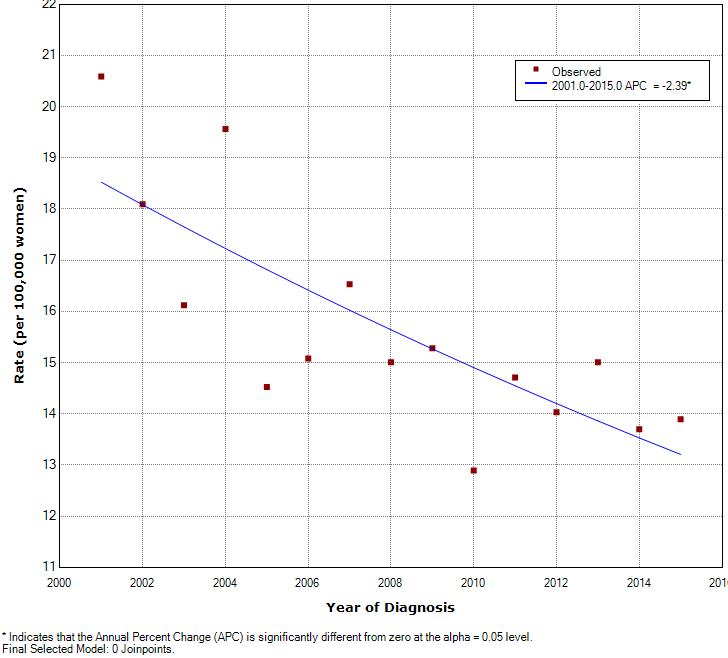

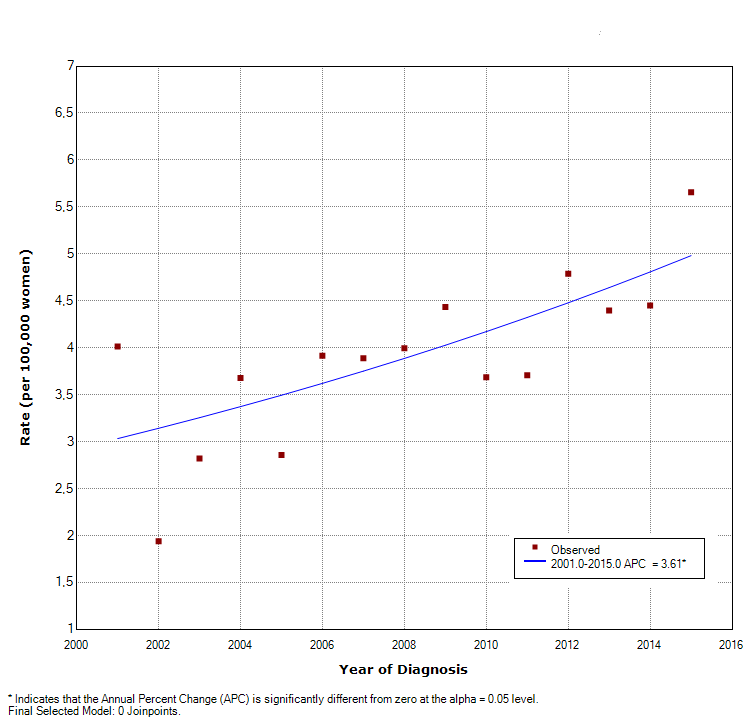

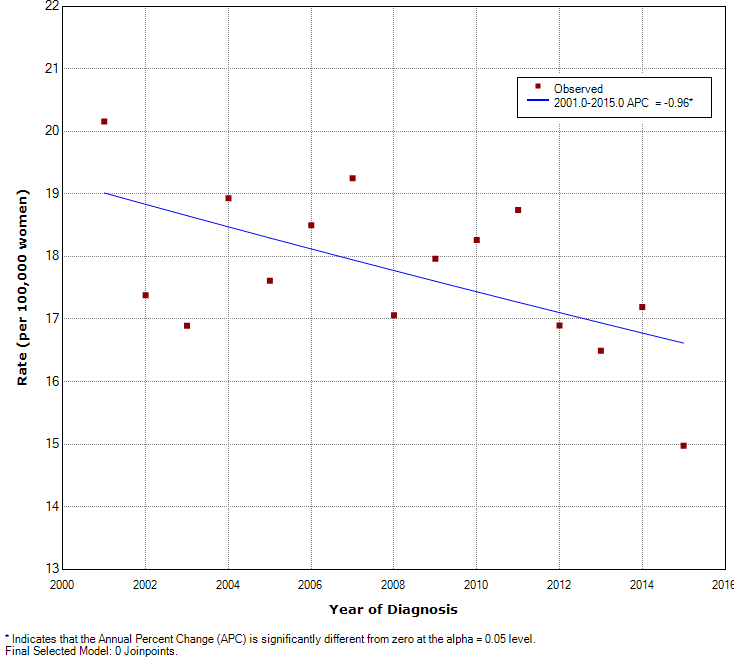

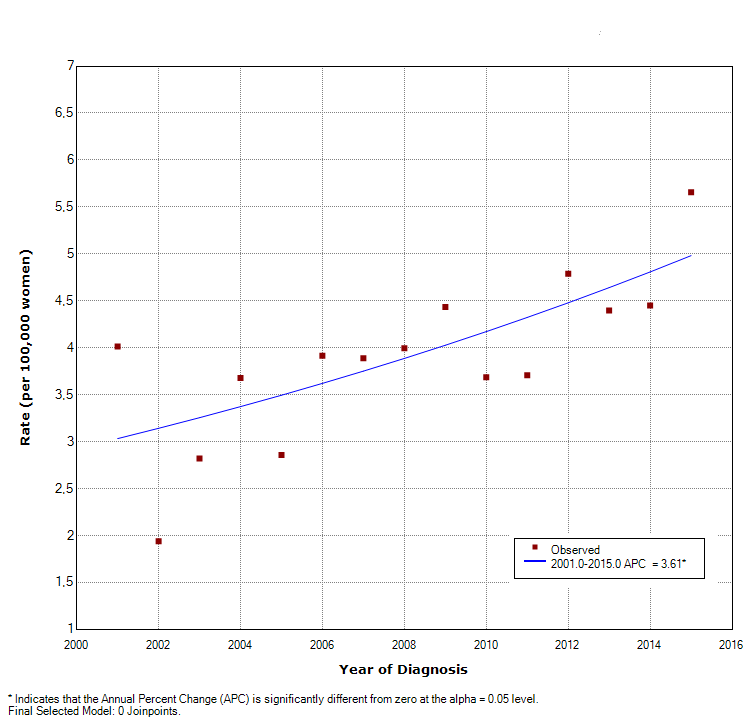

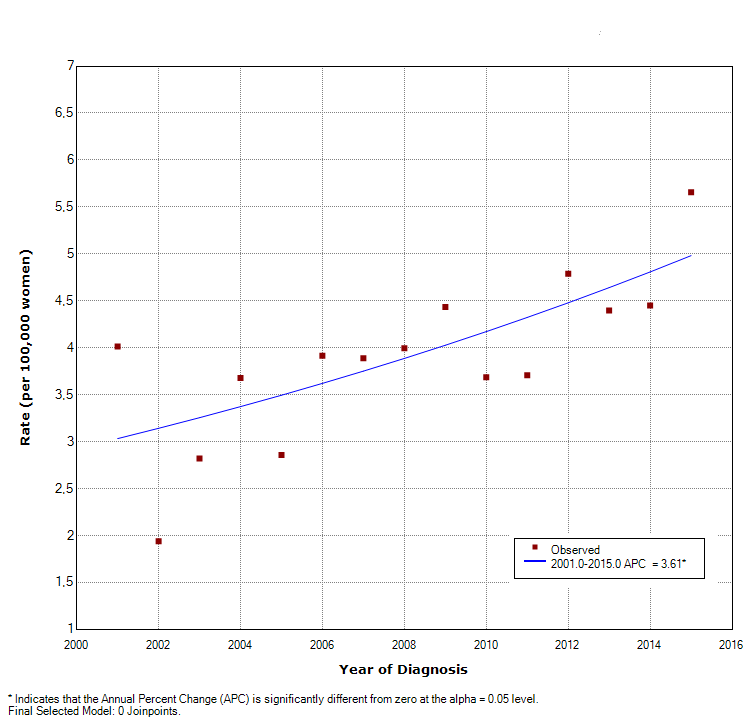

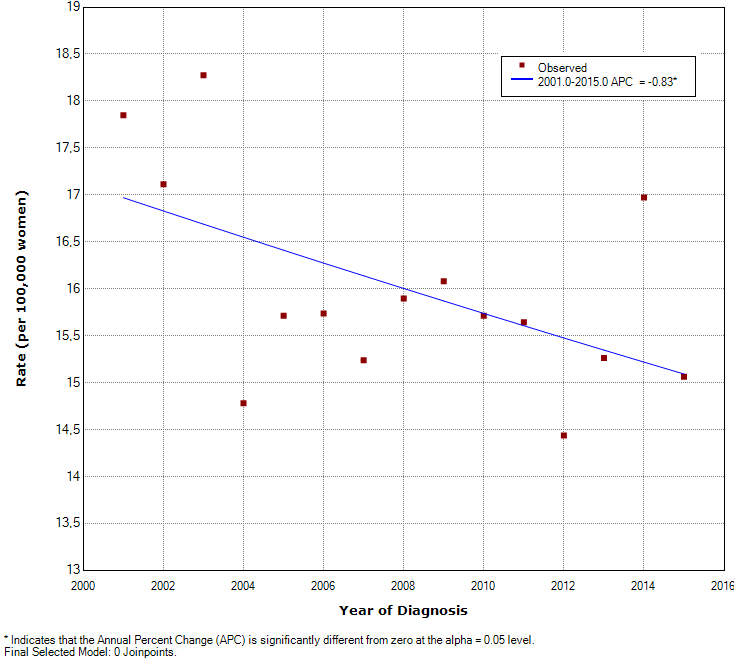

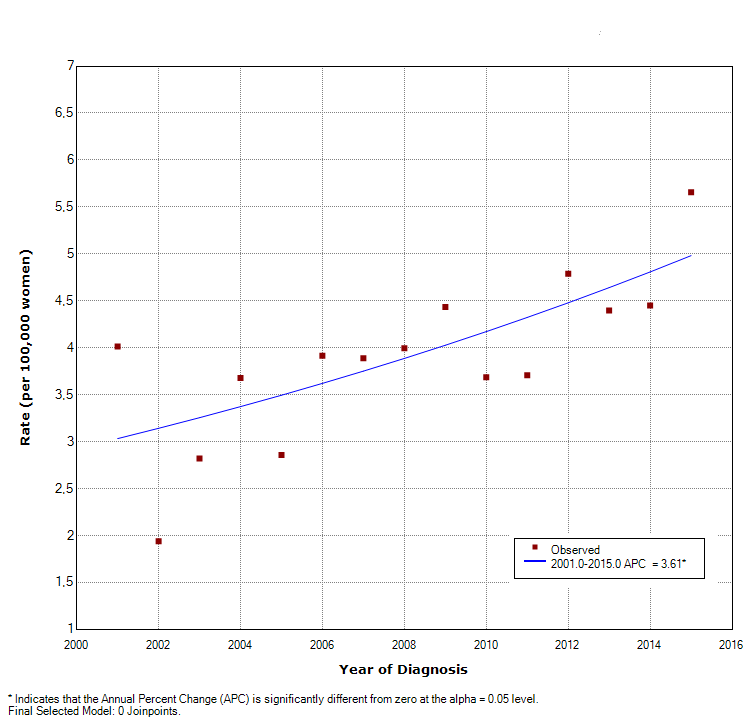


**C Age 40-49 years D Age 50-59 years**

**E Age 60-69 years F Age 70-79 years**

**Supplementary Figure 2:** Trends in incidence rates of cervical cancer (not corrected for hysterectomy) by age group; A shows trends in incidence for age group 20-29, B for 30-39, C for 40-49, D for 50-59, E for 60-69 and F for 70-79. * Indicates that the annual percent change (APC) is significantly different from zero at p-value<0.05. Final selected models for all age groups: 0 joinpoints.

**A Age 20-29 years B Age 30-39 years**


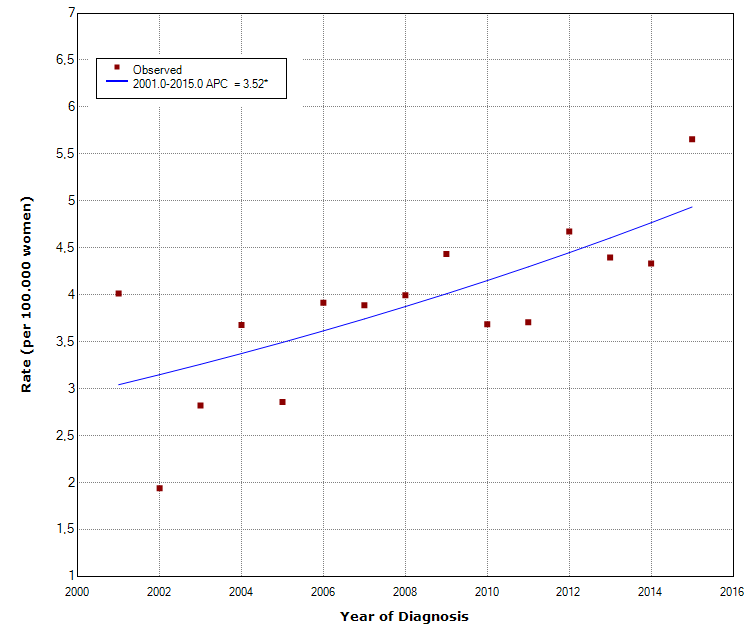

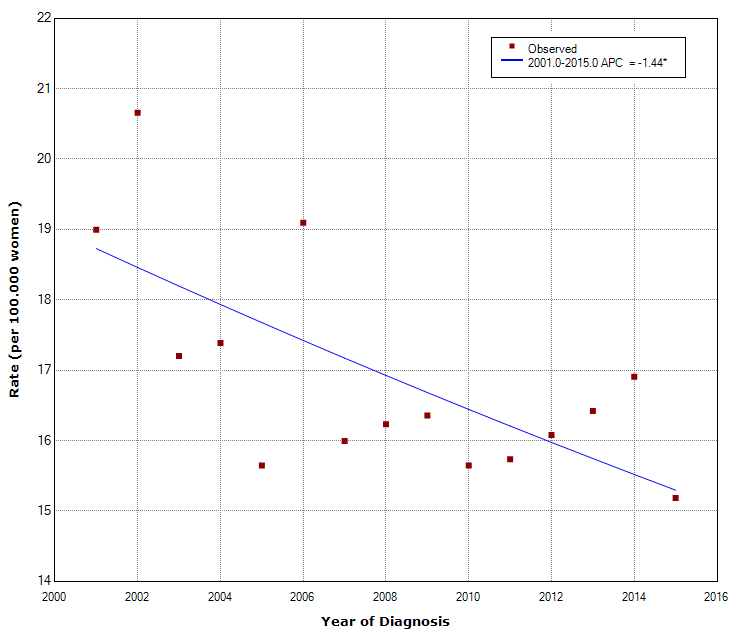

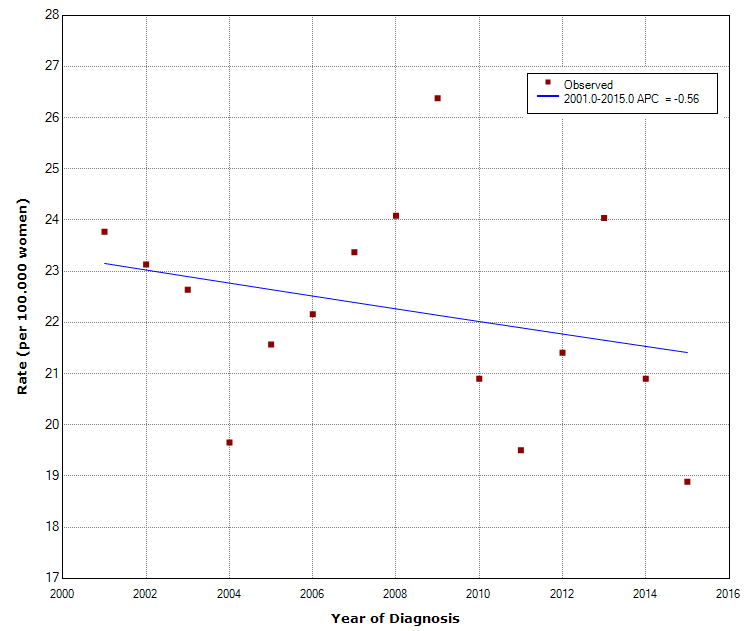

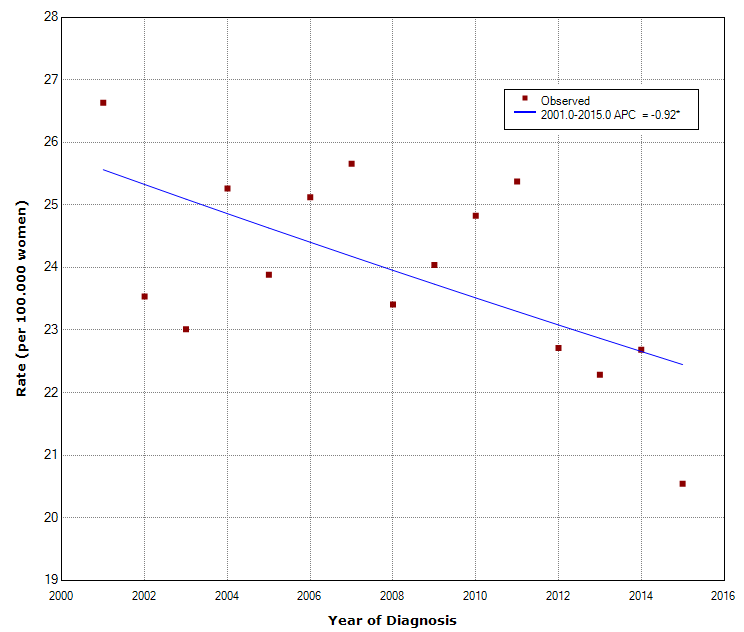

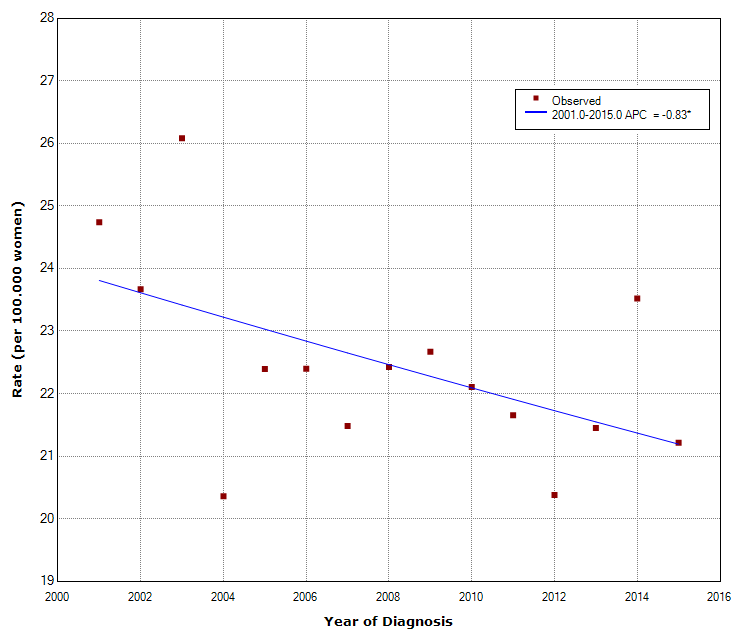

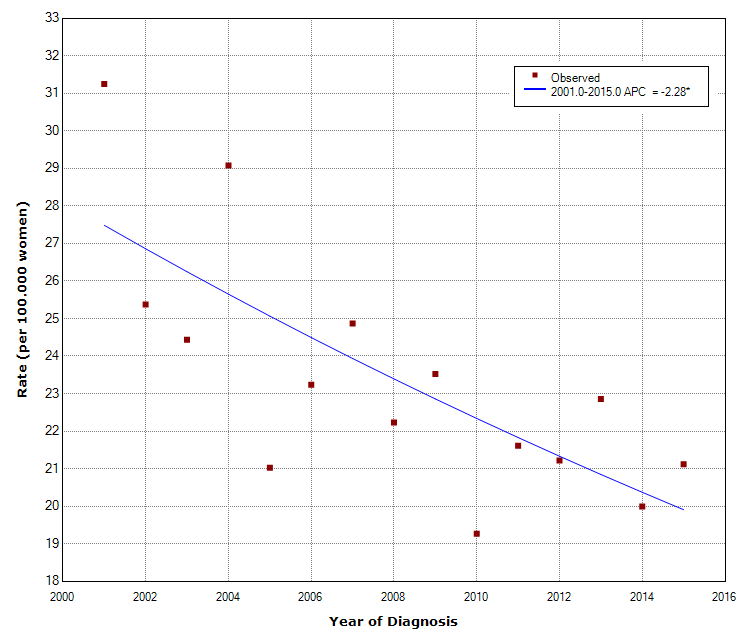


**C Age 40-49 years D Age 50-59 years**

**E Age 60-69 years F Age 70-79 years**

**Supplementary Figure 3:** Trends in hysterectomy corrected incidence rates of cervical cancer by age group (population at risk corrected using hysterectomy prevalence values by Prütz et al.); A shows trends in incidence for age group 20-29, B for 30-39, C for 40-49, D for 50-59, E for 60-69 and F for 70-79. * Indicates that the annual percent change (APC) is significantly different from zero at p-value<0.05. Final selected models for all age groups: 0 joinpoints.

**References**

1. Prütz F, Knopf H, Lippe Evd, Scheidt-Nave C, Starker A, Fuchs J. Prävalenz von hysterektomien bei frauen im alter von 18 bis 79 jahren: Robert Koch-Institut, Epidemiologie und Gesundheitsberichterstattung, 2013.

2. Zentrum für Krebsregisterdaten im Robert Koch-Institut. Datenbankabfrage mit schätzung der inzidenz, prävalenz und des überlebens von krebs in deutschland auf basis der epidemiologischen landeskrebsregisterdaten Available from URL: [www.krebsdaten.de/abfrage](file://nas.ads.mwn.de/ga83jev/Publikationen/Neumeyer%20CC%20in%20Elderly%20Women%20September%202021/2022%20April%20Cancer%20Medicine/1.%20revision%20Cancer%20Medicine/www.krebsdaten.de/abfrage) [accessed 22.10.2021, 2021].

3. Waterhouse J, Muir CS, Correa P, Powell J, eds. Cancer incidence in five continents. Lyon: IARC, 1976.

4. Day JC. Population projections of the united states by age, sex, race, and hispanic origin:1995 to 2050. Current Population Reports. Washington, DC: US Bureau of the Census, 1996:P25-1130.

5. Cancer incidence in five continents: A technical report. New York: Springer-Verlag, 1966.
